# Phosphorylation of Rab-coupling protein by LMTK3 controls Rab14-dependent EphA2 trafficking to promote cell:cell repulsion

**DOI:** 10.1038/ncomms14646

**Published:** 2017-03-15

**Authors:** Christine Gundry, Sergi Marco, Elena Rainero, Bryan Miller, Emmanuel Dornier, Louise Mitchell, Patrick T. Caswell, Andrew D. Campbell, Anna Hogeweg, Owen J. Sansom, Jennifer P. Morton, Jim C. Norman

**Affiliations:** 1 CRUK Beatson Institute for Cancer Research, Garscube Estate, Glasgow G61 1BD, UK; 2 Institute of Cancer Sciences, University of Glasgow, Glasgow G61 1QH, UK; 3 Cell-Matrix Research, Faculty of Life Sciences, University of Manchester, Manchester M13 9PT, UK

## Abstract

The Rab GTPase effector, Rab-coupling protein (RCP) is known to promote invasive behaviour *in vitro* by controlling integrin and receptor tyrosine kinase (RTK) trafficking, but how RCP influences metastasis *in vivo* is unclear. Here we identify an RTK of the Eph family, EphA2, to be a cargo of an RCP-regulated endocytic pathway which controls cell:cell repulsion and metastasis *in vivo*. Phosphorylation of RCP at Ser^435^ by Lemur tyrosine kinase-3 (LMTK3) and of EphA2 at Ser^897^ by Akt are both necessary to promote Rab14-dependent (and Rab11-independent) trafficking of EphA2 which generates cell:cell repulsion events that drive tumour cells apart. Genetic disruption of RCP or EphA2 opposes cell:cell repulsion and metastasis in an autochthonous mouse model of pancreatic adenocarcinoma—whereas conditional knockout of another RCP cargo, α5 integrin, does not suppress pancreatic cancer metastasis—indicating a role for RCP-dependent trafficking of an Eph receptor to drive tumour dissemination *in vivo*.

## Abstract (web summary)

Ephrin receptors mediate contact inhibition, but their intracellular trafficking during this process is unknown. Here the authors show that EphA2 receptor trafficking is regulated by the Rab GTPase effector Rab-coupling protein, which associates with Rab14-endosomes upon LMTK3-mediated phosphorylation.

The Rab family of GTPases and their effector proteins, which control endosomal trafficking, often have altered expression in tumours and, in some cases, this can drive tumour aggressiveness^1^,^2^,^3^,^4^. The Rab11 effector, Rab-coupling protein (RCP) is a component of the 8p11–12 amplicon which is commonly amplified in luminal-type breast cancers, and increased RCP expression has been shown to drive aggressiveness in this tumour type^5^. Furthermore, RCP is expressed at elevated levels in head and neck squamous cell carcinoma^6^, colon cancers^4^ and non-small cell lung carcinoma^7^. RCP regulates transferrin trafficking^8^ and in macrophages is thought to control recycling from phagosomes^9^. More recent work has shown that mutant p53 drives invasive migration of cancer cells by activating RCP-dependent trafficking of α5β1 integrin and receptor tyrosine kinases^10^,^11^.

The capacity of cells to repel one another is likely to contribute to cell dissemination in both normal and pathophysiological situations. Indeed, it is now considered likely that during metastasis homotypic repulsion between cancer cells is promoted—encouraging cancer cells to move away from the primary tumour and disperse, while heterotypic repulsion between cancer and non-cancerous cells is suppressed—allowing cancer cells to migrate into the surrounding tissue^12^,^13^,^14^,^15^. Repulsive cues are normally initiated by engagement of particular cell surface receptors with their cognate ligands situated at the plasma membrane of neighbouring cells. Many receptor:ligand pairs that mediate cell:cell repulsion are upregulated and/or modified in cancer. A recent large-scale exon sequencing and copy number analysis has indicated that ligand:receptor pairs of the semaphorin:plexin and ephrin:Eph families are associated with aggressiveness of pancreatic adenocarcinoma (PDAC)^16^. Although there is some dispute over the role of Ephs and ephrins in early tumorigenesis, it is generally thought that Ephs, in particular EphA2, are drivers of invasion and metastasis. In oesophageal squamous cell carcinoma^17^ and breast cancer^18^ high EphA2 expression correlates with poor prognosis, and EphA2 knockout reduces tumour growth and metastasis in ErbB2-driven breast cancer in mice. More recently Eph:ephrin interaction has been found to initiate cell:cell repulsion in prostate cancer cells in a way that would be expected to drive invasion and metastasis^19^. As with other receptor tyrosine kinases, it is likely that Eph receptor function is influenced by trafficking through the endosomal system. Indeed, one recent study reports that EphA2 is regulated by proteolytic cleavage at the cell surface, which allows the receptor to be internalized to activate signalling responses at endosomal membranes^20^.

Here, we identify a novel pathway in which a transmembrane serine/threonine kinase (LMTK3) phosphorylates a Rab effector (RCP) to control EphA2 trafficking. This initiates EphA2-mediated cell:cell repulsion, thus enabling cancer cells to move away from each other. In parallel with this we show that RCP and EphA2 are required for efficient invasion and metastasis in an *in vivo* model of PDAC suggesting that cell-cell repulsion is an important step in the metastatic spread of cancer.

## Results

### EphA2 associates with RCP

**Figure 1:**
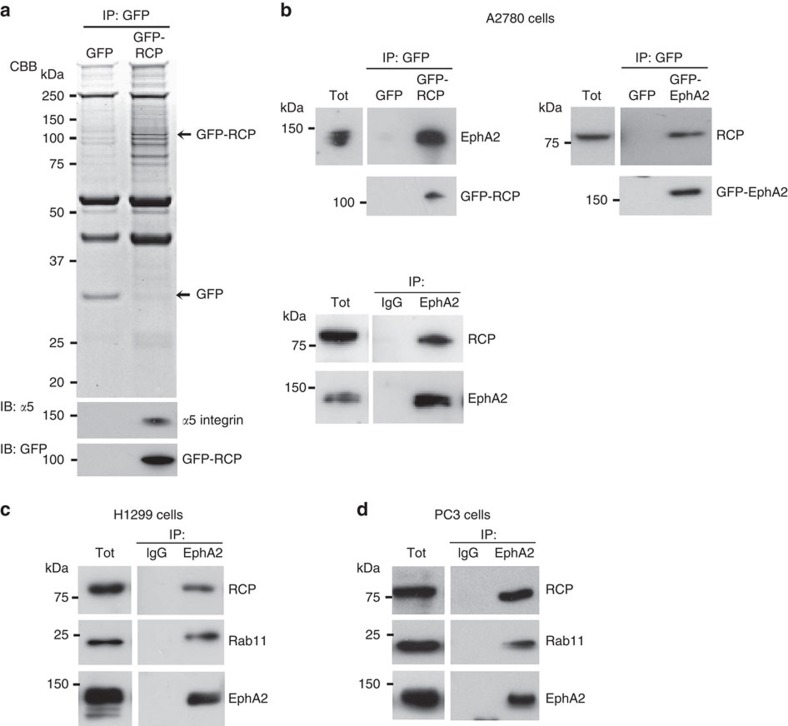
EphA2 associates with RCP. (**a**) A2780 cells expressing GFP-RCP or GFP control were lysed in a buffer containing 0.15% Tween-20. GFP was immunoprecipitated from lysates using magnetic beads conjugated to an antibody recognizing GFP. Immunoprecipitated proteins were separated by SDS-PAGE. and visualized using Coomassie brilliant blue (CBB). GFP-RCP and α5-integrin were detected by immunoblotting (IB) as indicated. (**b**) A2780 cells were transfected with GFP and GFP-RCP or EphA2-GFP (upper panels) or were left untransfected (lower panel). Cells were lysed in a buffer containing 0.15% Tween-20 and lysates were incubated with magnetic beads conjugated to antibodies recognizing GFP (upper panels) or EphA2 (lower panel). The immunoprecipitates were analysed by immunoblotting using antibodies recognizing RCP and EphA2. (**c**,**d**) EphA2 was immunoprecipitated from H1299 (**c**) and PC3 (**d**) cells as for (**b**) and the immunoprecipitates analysed by immunoblotting for RCP, Rab11 and EphA2. Uncropped blots corresponding to the experiments presented in **c**,**d** are displayed in Supplementary Fig. 8.

To search for new cargoes of endocytic recycling pathways that drive cancer cell migration and invasion, we screened RCP's interactome using immunoprecipitation and mass spectrometry. A number of proteins specifically associated with RCP and these included known interactors, such as α5β1 integrin, EGFR1, Rab11 and Rab14, but also receptors that may represent new RCP cargoes (Fig. 1a,Table 1). EphA2 was abundant in RCP immunoprecipitates, and we confirmed its co-precipitation with endogenous and GFP-tagged RCP (irrespective of whether EphA2 or RCP was the bait), in a range of cancer cell lines (Fig. 1b-d).

### RCP is required for efficient cell repulsion

**Figure 2:**
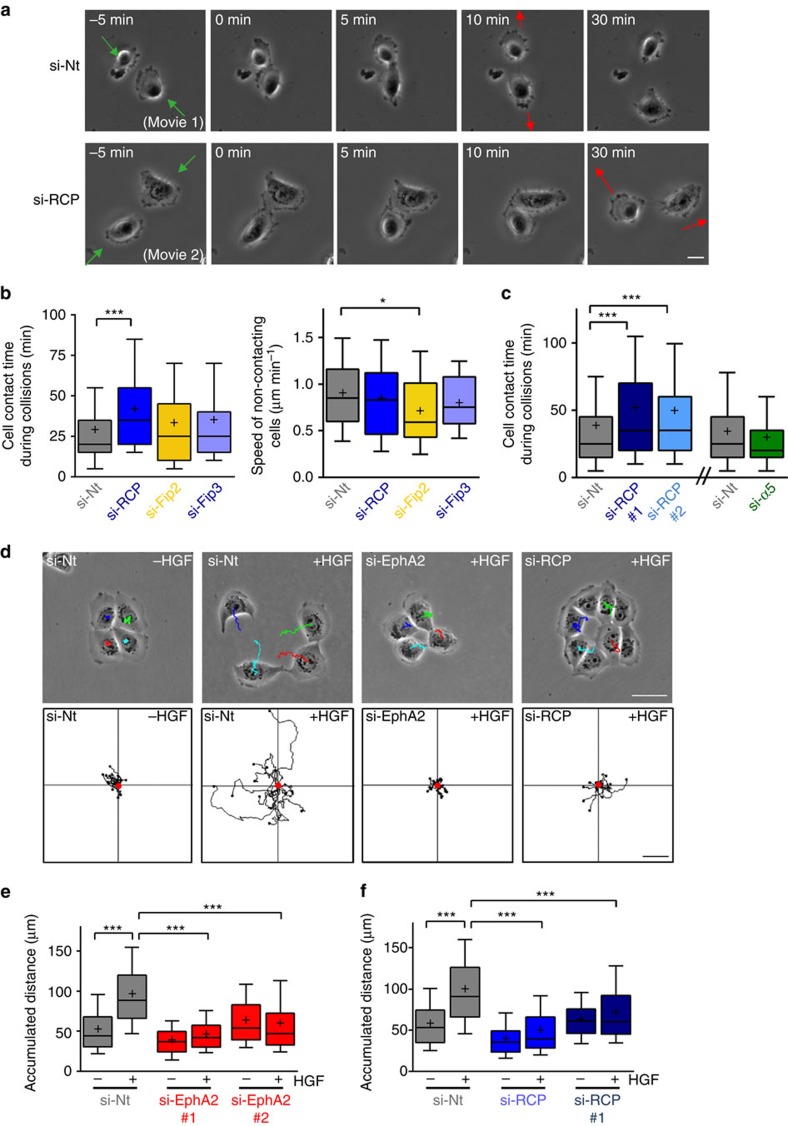
RCP and EphA2 influence cell:cell repulsion. (**a**–**c**) PC3 cells were transfected with siRNAs targeting RCP (SMARTPool (si-RCP) or two individual oligos (si-RCP#1 and si-RCP#2), FIP2 (si-FIP2), FIP3 (si-FIP3), α5 integrin (si-α5) or a non-targeting control (si-Nt). Transfected cells were sparsely seeded onto glass-bottomed wells coated with 33% Matrigel. Cells were serum-starved for 24 h and then treated with HGF (10 ng ml^−1^) to initiate cell migration. Cell movement was recorded using time-lapse video microscopy with frames being collected every 5 min. Representative movies illustrate collisions between control (si-Nt; movie 1) and RCP knockdown (si-RCP; movie 2) cells and frames from these are displayed in **a**. The frames have been aligned so that the start of the cell collision is at *t*=0 min. The green arrows indicate the direction from which the cells approached one another, and the red arrows indicate the direction of migration after the collision. The time that cells spent touching each other during collision was determined for all collisions in which only two cells were involved and both cells were migrating towards each other pre-collision (**b**; left panel and **c**). The speed of cell migration between collisions was also calculated from these movies (**b**; right panel). Bar, 10 μm. Data are represented as box and whiskers plots (whiskers: 10–90 percentile,+represents the mean). ***P<0.001, *P<0.01; one-way ANOVA (Kruskal-Wallis test, Dunn's Multiple Comparison Test). Data are from three independent experiments with >50 collisions being tracked per condition. (**d**–**f**) H1299 cells were transfected with siRNAs targeting RCP (SMARTPool (si-RCP) or an individual oligo (si-RCP#1), individual oligos targeting EphA2 (EphA2#1 and EphA2#2) or a non-targeting control (si-Nt). Transfected cells were seeded onto plastic surfaces and allowed to grow overnight to form colonies of approx. 4 cells per colony. Cells were then treated with HGF (10 ng ml^−1^) and cell scattering was recorded using time-lapse video microscopy with frames being collected every 5 min over a 6 h period. Representative trackplots of movies illustrate the scattering reaction of control (si-Nt), EphA2 knockdown (si-EphA2) and RCP knockdown (si-RCP) cells (**d**). Cell scattering was quantified using ImageJ manual tracking and chemotaxis plugin, and is expressed as the accumulated distance travelled over 6 h (**e**,**f**). Data are represented as box and whiskers plots (whiskers: 10–90 percentile,+represents the mean). ***P<0.001; one-way ANOVA (Kruskal-Wallis test, Dunn's Multiple Comparison Test). Data are from three independent experiments with >50 cells being tracked per condition. Bar in **d**, 50 μm.

Given EphA2's association with RCP, we determined the requirement for Rab GTPase-controlled endosomal trafficking in cell repulsion. When PC3 cells are treated with hepatocyte growth factor (HGF), they migrate rapidly on Matrigel-coated surfaces and numerous examples of cell repulsion may be observed. For example, in Fig. 2a, two PC3 cells migrated towards one another (green arrows), and upon collision (0 min) they stopped migrating for several minutes (Supplementary Movies 1 and 2). The cells then migrated away from each other in a direction that was different from their approach (red arrow). Quantification of a large number of collisions indicated that RCP knockdown significantly increased the time that colliding cells remained in contact before migrating away from one another, while siRNA of other Rab11 effectors (Fip2 or Fip3) or RCP interactors (α5β1 integrin) was ineffective in this regard (Fig. 2b,c; Supplementary Fig. 1a). Moreover, siRNA of RCP reduced the difference between the direction of cell migration before and after the collision, indicating the decreased tendency of RCP knockdown cells to change direction following collisions (Fig. 2a; Supplementary Movies 1 and 2). RCP knockdown did not affect migration speed of non-colliding cells (Fig. 2b), indicating that this Rab effector plays a role in cell repulsion in a manner which is distinct from the regulation of cell migration *per se*.

As cell:cell repulsion is likely to contribute to cancer cell dissemination, we tested the requirement for EphA2 and RCP in cell scattering. H1299 non-small cell lung cancer cells grow in tight colonies which scatter rapidly following addition of HGF (Fig. 2d). Time-lapse/cell tracking indicated that knockdown of either EphA2 or RCP (Fig. 2d–f; Supplementary Fig. 1b) strongly suppressed cell scattering. Interestingly, HGF-driven migration of sparsely plated non-contacting H1299 cells was not influenced by knockdown of RCP or EphA2 (Supplementary Fig. 2a) indicating that the cell migratory machinery was not, *per se*, inhibited by EphA2 or RCP knockdown and demonstrating that the role of these proteins in cell scattering is owing to their contribution to cell:cell repulsion.

### RCP and Rab14 control EphA2 trafficking and function

**Figure 3:**
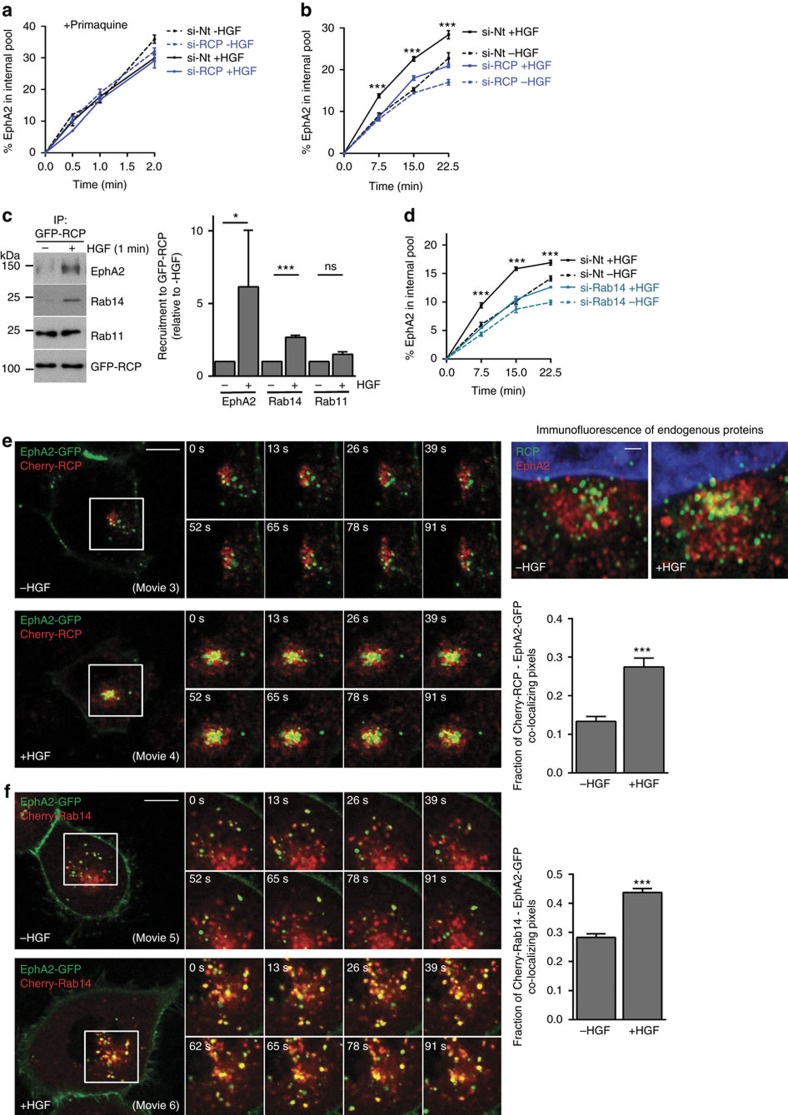
HGF-driven trafficking of EphA2 is controlled by RCP and Rab14. (**a**,**b**) H1299 cells were transfected with siRNAs targeting RCP (si-RCP) or a non-targeting control (si-Nt) and plated onto 10 cm plastic dishes. Cells were surface-labelled with NHS-S-S-Biotin (0.13 mg ml^−1^) at 4 °C and internalization allowed to proceed at 37 °C for the indicated times in the presence (**a**) or absence (**b**) of primaquine (0.6 mM), with or without HGF (10 ng ml^−1^). Biotin remaining at the cell surface was removed by exposure to MesNa at 4 °C and the quantity of biotinylated receptors within the cells determined by capture-ELISA using microtitre wells coated with monoclonal antibodies recognizing EphA2. Values are mean±s.e.m. from either one representative experiment performed in quadruplicate (**a**) or from three independent experiments (**b**), ***P<0.001 (si-RCP+HGF versus si-Nt+HGF); two-way ANOVA, Bonferroni post-test. (**c**) H1299 cells expressing GFP-RCP were incubated in the presence or absence of HGF (10 ng ml^−1^) for 1 min. Cells were lysed in a buffer containing 0.15% Tween-20 and GFP was immunoprecipitated as for Fig. 1c. The immunoprecipitates were analysed by immunoblotting using antibodies recognizing EphA2, Rab14, Rab11 and RCP. Recruitment of EphA2, Rab14 and Rab11 to GFP-RCP immunoprecipitates was quantified by densitometric scanning of western blots. Values are mean±s.e.m. from three independent experiments; *P<0.05, ***P<0.001, ns, not significant; Mann-Whitney test. Uncropped blots corresponding to the experiment presented in Fig. 3c are displayed in Supplementary Fig. 9. (**d**) H1299 cells were transfected with siRNAs targeting Rab14 (si-Rab14) or a non-targeting control (si-Nt) and internalization of EphA2 in the presence and absence of HGF was determined as for **b**. Values are mean±s.e.m. from three independent experiments, ***P<0.001 (si-Rab14+HGF versus si-Nt+HGF); two-way ANOVA, Bonferroni post-test. (**e**,**f**) H1299 cells were transfected with EphA2-GFP in combination with either mCherry-RCP (**e**) or mCherry-Rab14 (**f**) and plated onto glass-bottomed dishes, or were left untransfected. Twenty-four hours following transfection, confocal time-lapse movies were collected with 2 s frame intervals over a 2 min period in the presence and absence of HGF (10 ng ml^−1^). HGF was added 30 min before collecting the movies. Stills were extracted from movies 3-4 at the indicated time points following collection of the first frame of the movie and display the region of interest indicated by the white boxes. Bar, 10 μm. Olympus software was used to quantify co-localized pixels relative to the EphA2-GFP and mCherry-RCP pixels across the course of the movies, and these are plotted as the fraction of co-localizing pixels as determined by the Costes method^39^. Values are mean±s.e.m. from three separate experiments incorporating >60 cells per condition, ***P<0.001 Mann-Whitney test. In the right panels in **e** untransfected H1299 cells were incubated in the presence or absence of HGF (10 ng ml^−1^) for 30 min and then fixed. Endogenous RCP and EphA2 were visualized by immunofluorescence with respect to the cell nucleus (stained with DAPI) following by high resolution imaging using a high resolution Airy-scan microscope. Detail of the perinuclear region is displayed. Bar, 1 μm. The whole cell fluorescence micrographs and single channel fluorescence images of these Airy-scan images are presented in Supplementary Fig. 4a.

To obtain estimates of EphA2 internalization that were unaffected by receptor recycling, we performed endocytosis assays in the presence of the recycling inhibitor, primaquine. This indicated that neither HGF-addition nor RCP-knockdown affected EphA2 endocytosis *per se* (Fig. 3a). In the absence of primaquine, however, addition of HGF significantly enhanced the rate of EphA2 accumulation within H1299 cells (Fig. 3b). These data indicate that HGF does not affect EphA2 endocytosis, but reduces the rate at which internalized EphA2 recycles to the cell surface. Importantly, HGF's ability to promote intracellular accumulation of EphA2 was partially reversed by RCP knockdown (Fig. 3b).

HGF-addition significantly increased the quantity of EphA2 that coimmunoprecipitated with RCP, consistent with a functional role for RCP in EphA2 trafficking (Fig. 3c). RCP can bind to Rab14 as well as Rab11 (Table 1) (refs 21, 22), so we probed RCP immunoprecipitates with antibodies recognizing these Rab GTPases. HGF promoted association of Rab14 with this complex, suggesting that Rab14 may contribute to EphA2 trafficking (Fig. 3c). By contrast, the amount of Rab11 which coimmunoprecipitated with RCP was not reproducibly increased by addition of HGF (Fig. 3c). Knockdown of Rab14 (but not Rab11) completely opposed HGF-driven cellular accumulation of EphA2, indicating that Rab14 is necessary for RCP-mediated EphA2 trafficking (Fig. 3d; Supplementary Fig. 2b).

We expressed EphA2-GFP with mCherry-RCP or mCherry-Rab14 and monitored the trafficking of these proteins in the presence and absence of HGF. EphA2-GFP was localized to the plasma membrane and intracellular vesicles that were in constant motion and were frequently seen near cell:cell contacts (Fig. 3e; Supplementary Movie 3). By contrast, mCherry-RCP and mCherry-Rab14 were localized to the peri-nuclear region, and there was little co-localization between EphA2-GFP and mCherry-RCP or mCherry-Rab14 (Fig. 3e,f; Supplementary Movies 3 and 5). Following addition of HGF, EphA2-GFP was relocated to RCP- and Rab14-positive structures, and quantitative analysis indicated that this was statistically significant across a number of experiments (Fig. 3e,f; Supplementary Movies 4 and 6). Furthermore, high resolution imaging of fixed cells indicated that both EphA2-GFP and mCherry-RCP, and endogenous EphA2 and RCP are present in a subset of closely apposed perinuclear endosomes that possess both overlapping and non-overlapping domains (Fig. 3e; Supplementary Fig. 3a,b).

**Figure 4:**
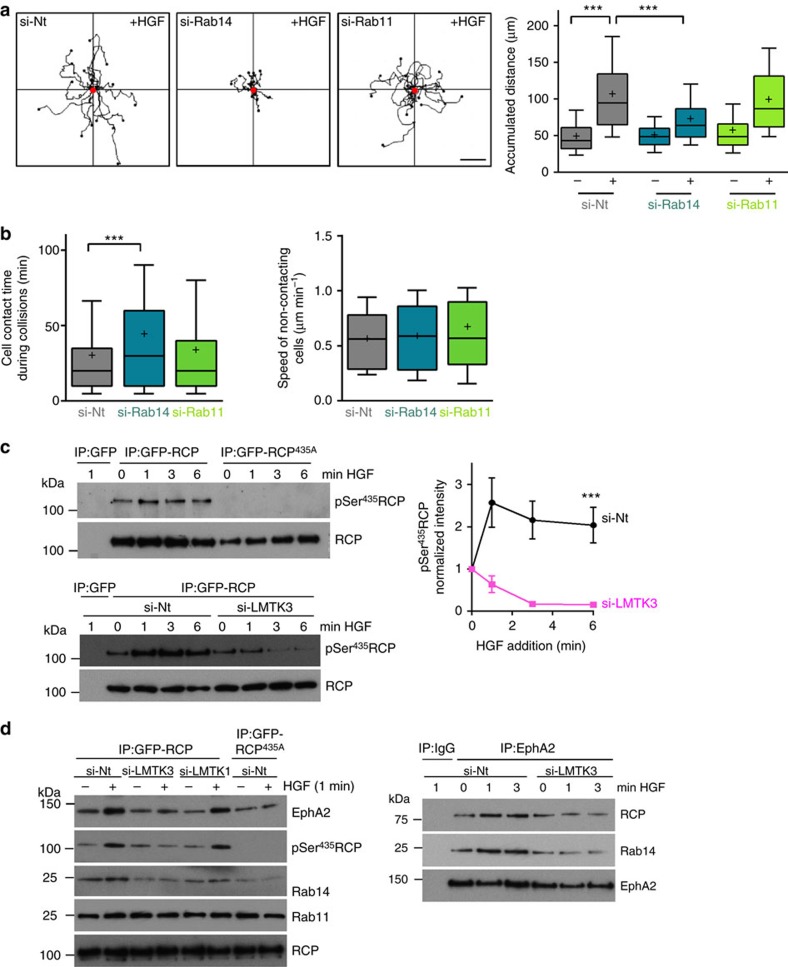
Rab14 controls cell repulsion and LMTK3 phosphorylates RCP at Ser^435^. (**a**) H1299 cells were transfected with siRNAs targeting Rab14 (si-Rab14), Rab11 (si-Rab11) or a non-targeting control (si-Nt) and cell scattering was determined as for Fig. 2d,e. Representative trackplots from these experiments are displayed and cell scattering was quantified as for Fig. 2e and is expressed as the accumulated distance travelled over 6 h. Data are represented as box and whiskers plots (whiskers: 10–90 percentile,+represents the mean). ***P<0.001; one-way ANOVA (Kruskal-Wallis test, Dunn's Multiple Comparison Test). Data are from three independent experiments with >50 cells being tracked per condition. Bar, 50 μm. (**b**) PC3 cells were transfected with siRNAs targeting Rab14, Rab11 or a non-targeting control (si-Nt) and the contact time during collisions (left panel) and migration speed between collisions (right panel) was determined as for Fig. 2a,b. Data are represented as box and whiskers plots (whiskers: 10–90 percentile,+represents the mean). ***P<0.001; one-way ANOVA (Kruskal-Wallis test, Dunn's Multiple Comparison Test). Data are from three independent experiments with >50 collisions being tracked per condition. (**c**,**d**) H1299 cells were transfected with GFP, GFP-RCP or GFP-RCP^435A^ in combination with siRNAs targeting LMTK3 (si-LMTK3), LMTK1 (si-LMTK1) or non-targeting control (si-Nt) and plated onto 15 cm plastic dishes. In the right panel of **d** cells were transfected with siRNA targeting LMTK3 (si-LMTK3) or non-targeting control in the absence of GFP or GFP-tagged RCPs. Forty-eight hours following transfection, cells were treated with HGF (10 ng ml^−1^) for the indicated times or were left untreated (0 min). Cells were lysed in a buffer containing 0.15% Tween-20 and GFP or EphA2 immunoprecipitated (IP) as for Fig. 1c. The immunoprecipitates were analysed by immunoblotting using antibodies recognizing EphA2, Rab14, Rab11, phosphoSer^435^RCP and RCP. The quantity of phosphoSer^435^RCP in the immunoblots was estimated by densitometric scanning (**c**; right panel). Data are mean±s.e.m. from six independent experiments. ***P<0.001, Mann-Whitney test. Uncropped blots corresponding to the experiments presented in **c**,**d** are displayed in Supplementary Fig. 9.

These findings prompted us to investigate the respective requirements for Rab14 and Rab11 in EphA2-dependent cell:cell repulsion and scattering. siRNA of Rab14 opposed HGF-driven cell scattering, whereas knockdown of Rab11 was ineffective in this regard (Fig. 4a). Consistently, knockdown of Rab14 increased the time that colliding PC3 cells remained in contact before migrating away from one another, while siRNA of Rab11 did not (Fig. 4b; Supplementary Fig. 1a). Moreover, siRNA of Rab14 did not affect the migration speed of non-contacting cells. Taken together, these data indicate that signalling events downstream of HGF promote assembly of an RCP-Rab14 complex which controls EphA2 trafficking to enable cell:cell repulsion, and that RCP-Rab11 interaction is not required for this to occur.

### LMTK3 phosphorylates RCP to promote cell scattering

As HGF signalling may control RCP function by influencing its phosphorylation, we used mass spectrometry to identify residues in RCP that might function as phospho-acceptors. The most abundant phospho-peptides in RCP were ^430^ESRRSS_(HPO3)_LLSLMTGK^443^ and ^523^RPPISS_(HPO3)_PRAPQTRA^537^ (Supplementary Fig. 4a), and we raised a phospho-specific antibody against a synthetic peptide corresponding to one of these RCP^427-439^(pSer^435^) (Supplementary Fig. 5b). Addition of staurosporine led to substantial reduction in the signal detected by anti-pSer^435^-RCP (Supplementary Fig. 4b) indicating that RCP phosphorylation is a dynamic event and therefore potentially regulated. Indeed, increased phosphorylation of RCP at Ser^435^ was detected shortly following addition of HGF to H1299 cells (Fig. 4c).

The region surrounding RCP's Ser^435^ conforms to a consensus sequence for phosphorylation by the transmembrane endosomal serine/threonine kinase, LMTK2 (ref. 23). The LMTK family comprises three members, and siRNA experiments indicated that LMTK3 is responsible for HGF-induced phosphorylation of RCP's Ser^435^. Indeed, siRNA of LMTK3 (Fig. 4c,d), but not LMTK1 (Fig. 4d) opposed HGF-driven phosphorylation of RCP at Ser^435^.

**Figure 5:**
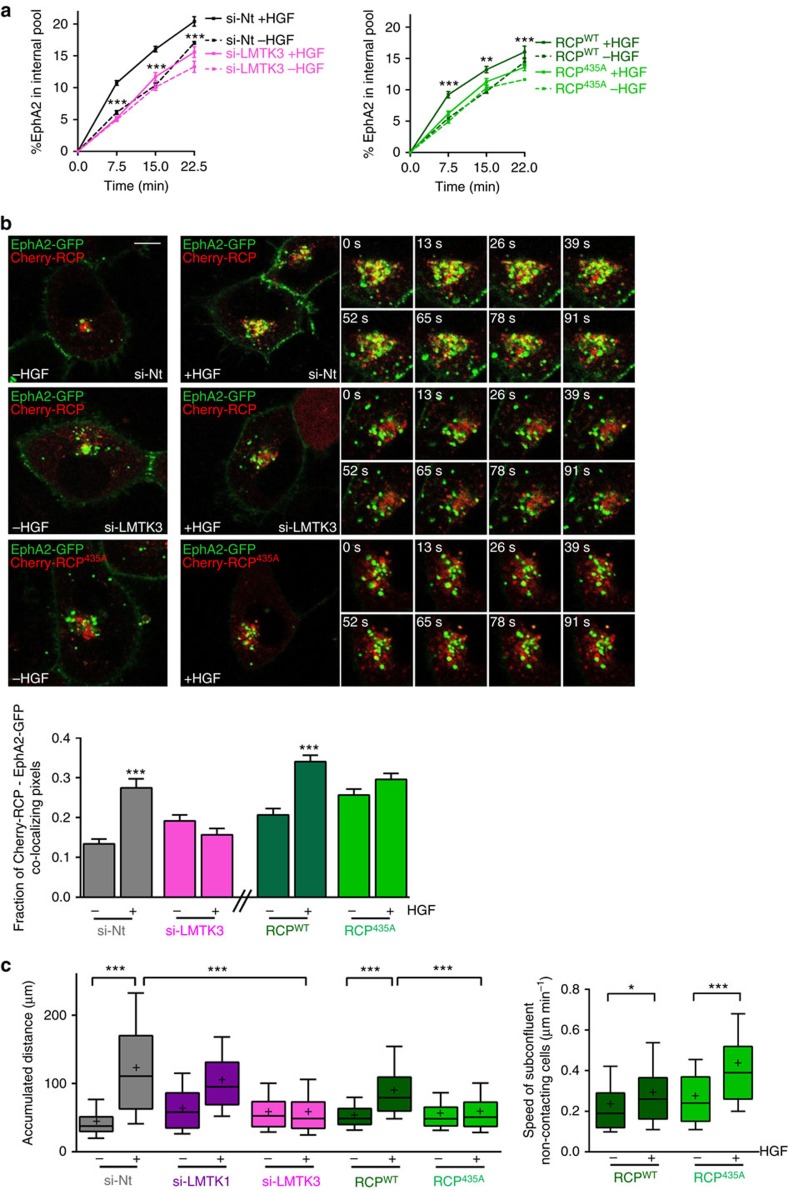
LMTK3 phosphorylation of RCP is necessary for HGF-driven EphA2 trafficking and cell scattering. (**a**) H1299 cells were transfected with siRNAs targeting LMTK3 (si-LMTK3) or a non-targeting control (si-Nt) (left panel). Alternatively cells were transfected with GFP-RCP or GFP-RCP^435A^ (right panel). Internalization of EphA2 in the presence and absence of HGF was then determined as for Fig. 3b. Values are mean±s.e.m. from three independent experiments. ***P<0.001 (si-LMTK3+HGF versus si-Nt+HGF) **P<0.01 (RCP^435A^+HGF versus RCP^WT^+HGF); two-way ANOVA, Bonferroni post-test. (**b**) H1299 cells were transfected with EphA2-GFP in combination with mCherry-RCP or mCherry-RCP^435A^, in the presence or absence of siRNAs targeting LMTK3 (si-LMTK3) or non-targeting control (si-Nt) as indicated. Trafficking of EphA2-GFP and mCherry-RCPs in the presence and absence of HGF (added 30 min before collecting the movies) was visualized by fluorescence confocal time-lapse microscopy and co-localization was quantified as for Fig. 3e. Stills were extracted from movies at the indicated time points following the collection of the first frame of the movie, and these display the region of interest indicated by the white box. Bar, 10 μm. Values are mean±s.e.m. from three separate experiments incorporating >60 cells per condition. ***P<0.001; one-way ANOVA (Kruskal-Wallis test, Dunn's Multiple Comparison Test). (**c**) H1299 cells were transfected with siRNAs targeting LMTK3 (si-LMTK3), LMTK1 (si-LMTK1) or a non-targeting control (si-Nt). Alternatively cells were transfected with GFP-RCP or GFP-RCP^435A^. Cell scattering in the presence and absence of HGF was then experimentally determined and quantified as for Fig. 2d,e. Scattering is expressed as the accumulated distance travelled by cells from their starting point over 6 h. In the right panel, GFP-RCP and GFP-RCP^435A^–expressing cells were plated subconfluently and the migration speed of non-contacting cells was determined in the presence and absence of HGF. Data are represented as box and whiskers plots (whiskers: 10–90 percentile,+represents the mean). ***P<0.001; one-way ANOVA (Kruskal-Wallis test, Dunn's Multiple Comparison Test). Data are from three independent experiments with >50 cells being tracked per condition.

siRNA of LMTK3 (but not LMTK1) or substitution of RCP's Ser^435^ with Ala opposed HGF-driven recruitment of RCP, Rab14 and EphA2 into a coimmunoprecipitatable complex (Fig. 4d). Moreover, following LMTK3 knockdown or expression of RCP^435A^, HGF did not promote intracellular accumulation of EphA2 (Fig. 5a). Consistently, quantitative live cell imaging and high resolution fluorescence microscopy indicated that HGF-driven transport of EphA2 to RCP-positive endosomes was opposed by siRNA of LMTK3 and by expression of mCherry-RCP^435A^ (Fig. 5b; Supplementary Fig. 3b). Taken together these data indicate that HGF signalling promotes LMTK3-mediated phosphorylation of RCP at Serine^435^ which leads to assembly of an RCP/Rab14/EphA2 complex and accumulation of EphA2 within perinuclear endosomes.

Having delineated signalling events leading to altered EphA2 trafficking, we then tested their role in cell repulsion and scattering. Disruption of LMTK3-mediated RCP phosphorylation, either by siRNA of LMTK3 or expression of RCP^435A^ opposed HGF-driven cell scattering (Fig. 5c), without suppressing the migration speed of non-contacting cells. By contrast, knockdown of LMTK1 was ineffective in this regard. Taken together these data identify a pathway linking HGF signalling to EphA2 trafficking which proceeds via LMTK3-mediated phosphorylation of RCP and recruitment of Rab14, and that this pathway must be intact in order for cells to activate the repulsive events that allow cells to move away from one another.

### Requirement for phospho-Ser^897^-EphA2 in cell scattering

EphA2 functions in ligand-dependent and -independent ways to influence cell movement. siRNA of ephrin-A1, the cognate ephrin ligand for EphA2 which is expressed by H1299 cells (Supplementary Fig. 5a), had no effect on cell scattering, indicating the possibility that engagement of EphA2 with its ephrin ligand might not be a key determinant of HGF-driven cell:cell repulsion. Ligand-engagement of EphA2 is known to promote phosphorylation of Tyr^588^ in its cytodomain (Supplementary Fig. 5b) which triggers ligand-dependent downstream signalling^24^. Consistently, occupation of EphA2 with ephrin-A1 drives complete redistribution of the receptor from the cell surface to endosomes, and this is opposed by substitution of Tyr^588^ with Phe (Supplementary Fig. 5c). However, mutation of EphA2's Tyr^588^ had no effect on HGF-driven intracellular accumulation of EphA2 and even increased the basal (ligand-independent) levels of receptor internalization (Supplementary Fig. 5d). Moreover, HGF promoted the delivery of EphA2-Tyr^588F^ to RCP-positive endosomes to a similar extent as seen for the wild-type receptor (Supplementary Fig. 5e) and, importantly, EphA2-Tyr^588F^ was fully capable of supporting HGF-driven cell scattering (Supplementary Fig. 5f).

**Figure 6:**
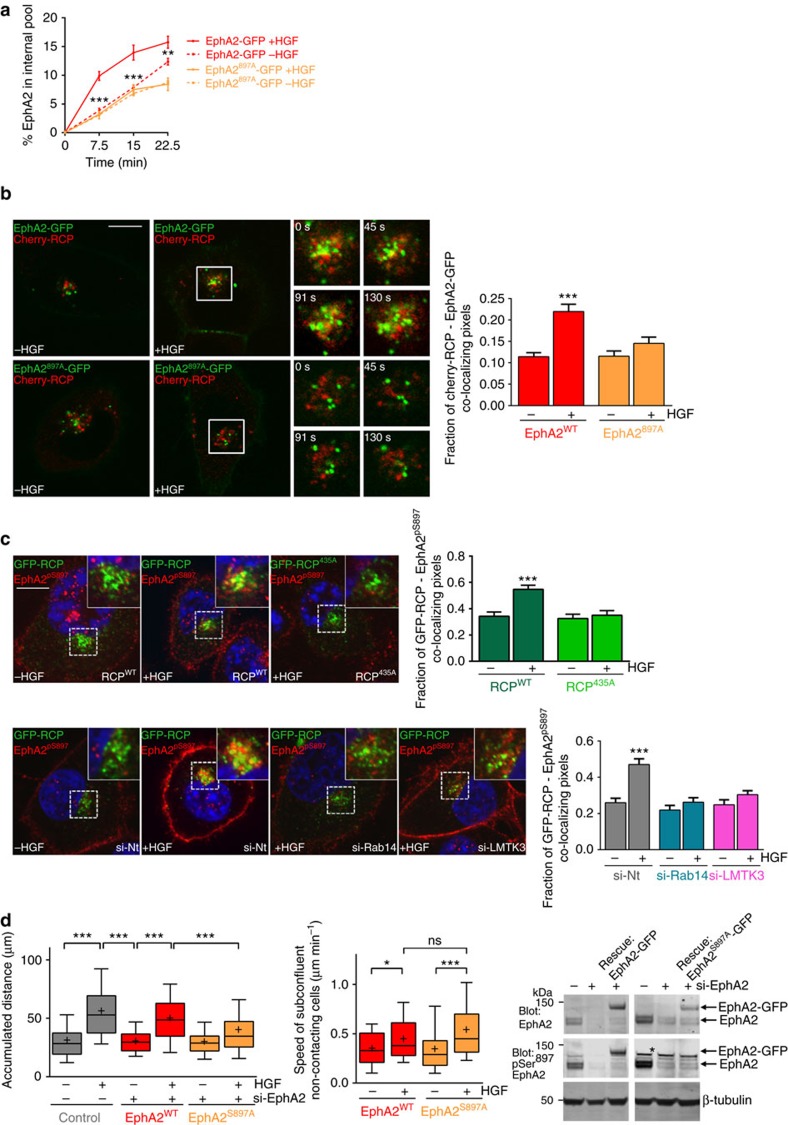
Phosphorylation of EphA2 at Ser^897^ is necessary for HGF-driven EphA2 trafficking and cell scattering. (**a**) H1299 cells were transfected with a siRNA targeting EphA2 in combination with siRNA-resistant forms of EphA2-GFP or EphA2^897A^-GFP. Internalization of EphA2-GFPs in the presence and absence of HGF was then determined as for Fig. 3b, but with the ELISA plate coated with anti-GFP to specifically detect only the GFP-tagged receptor. Values are mean±s.e.m. *n*=4 biological replicates from two independent experiments. ***P<0.001 (EphA2^897A^-GFP+HGF versus EphA2-GFP+HGF); two-way ANOVA, Bonferroni post-test. (**b**) H1299 cells were transfected with EphA2-GFP or EphA2^897A^-GFP in combination with mCherry-RCP. Trafficking of EphA2-GFP and mCherry-RCPs in the presence and absence of HGF (added 30 min before collecting the movies) was visualized by fluorescence confocal time-lapse microscopy and co-localization was quantified as for Fig. 3e. Stills were extracted from movies at the indicated time points, and these display the region of interest indicated by the white box. Bar, 10 μm. Values are mean±s.e.m. from three separate experiments incorporating >10 cells per condition per experiment. ***P<0.001; one-way ANOVA (Kruskal-Wallis test, Dunn's Multiple Comparison Test). (**c**) H1299 cells were transfected with GFP-RCP or GFP-RCP^435A^, in the presence or absence of siRNAs targeting Rab14 (si-Rab14), LMTK3 (si-LMTK3) or non-targeting control (si-Nt) as indicated. Cells were incubated in the presence or absence of HGF for 30 min and then fixed and phospho-Ser^897^-EphA2 (EphA2^pS897^) and nuclei were visualized by immunofluorescence followed by confocal microscopy. Bar, 10 μm. Co-localization of GFP-RCPs and EphA2^pS897^ was determined as for Fig. 3e. Values are mean±s.e.m. from three separate experiments incorporating >60 cells per condition. ***P<0.001; one-way ANOVA (Kruskal-Wallis test, Dunn's Multiple Comparison Test). (**d**) H1299 cells were transfected with a non-targeting siRNA or with an siRNA targeting EphA2 (si-EphA2) in combination with siRNA-resistant forms of EphA2-GFP or EphA2^897A^-GFP. Cell scattering and the migration of subconfluent, non-contacting cells in the presence and absence of HGF was then determined and quantified as for Fig. 5c. Data are represented as box and whiskers plots (whiskers: 10–90 percentile,+represents the mean). ***P<0.001; one-way ANOVA (Kruskal-Wallis test, Dunn's Multiple Comparison Test). Data are from three independent experiments with >50 cells being tracked per condition. The expression levels of the siRNA-resistant forms of EphA2-GFP and EphA2^897A^-GFP were determined in EphA2 knockdown cells by western blotting with antibodies recognizing EphA2 or pSer^897^-EphA2. The band marked with an asterisk is recognized non-specifically by the anti- pSer^897^-EphA2 antibody. Uncropped blots corresponding to the experiment presented in **d** are displayed in Supplementary Fig. 9.

Some functions of EphA2 are associated with phosphorylation of Ser^897^ which occurs following activation of Akt by growth factor receptors^25^. Consistent with previous reports, we found that HGF promoted phosphorylation of EphA2 on Ser^897^ (but not Tyr^588^) (Supplementary Fig. 6a). Moreover, knockdown of endogenous ephrin-A1 did not affect HGF-driven phosphorylation of EphA2's Ser^897^ (Supplementary Fig. 6a). To determine whether trafficking of EphA2 to the RCP/Rab14 compartment influences EphA2 phosphorylation, we knocked-down RCP, Rab14 or LMTK3 and measured levels of phospho-Ser^897^-EphA2 (EphA2^pS897^). However, siRNA of these regulators of EphA2 trafficking did not influence the cellular content of EphA2^pS897^ (Supplementary Fig. 6b). Furthermore, we have used a phosphoproteomic approach to map phosphorylated residues in EphA2's cytodomain and have not been able to identify residues other than Ser^897^ whose phosphorylation was influenced by HGF addition. We, therefore, investigated whether phosphorylation of EphA2 on Ser^897^ influences its endosomal trafficking. Substitution of Ser^897^ with Ala completely opposed HGF-driven intracellular accumulation of EphA2-GFP (Fig. 6a). Consistently, quantitative live cell imaging and high resolution microscopy demonstrated that EphA2^897A^-GFP was not trafficked to RCP-positive endosomes following HGF addition (Fig. 6b; Supplementary Fig. 3b). Furthermore, following HGF-addition, endogenous EphA2^pS897^ accumulated in RCP-positive endosomes in the perinuclear region of HGF-treated cells, and this was opposed by siRNA of LMTK3 or Rab14, or by expression of mCherry-RCP^435A^ (Fig. 6c; Supplementary Fig. 3c). Finally, although siRNA-resistant EphA2-GFP restored HGF-driven cell scattering in EphA2 knockdown cells, EphA2-GFP^897A^ was less effective in this regard (Fig. 6d). Importantly, despite EphA2-Ser^897A^'s inability to support cell scattering, this mutant of EphA2 did not oppose HGF-induced migration of non-contacting cells (Fig. 6d). Taken together, these data indicate that Akt-mediated phosphorylation of EphA2 at Ser^897A^ is required for its trafficking to RCP/Rab14 positive endosomes and for the consequent activation of cell repulsion.

### Requirement for RCP and EphA2 in metastasis of PDAC

**Figure 7:**
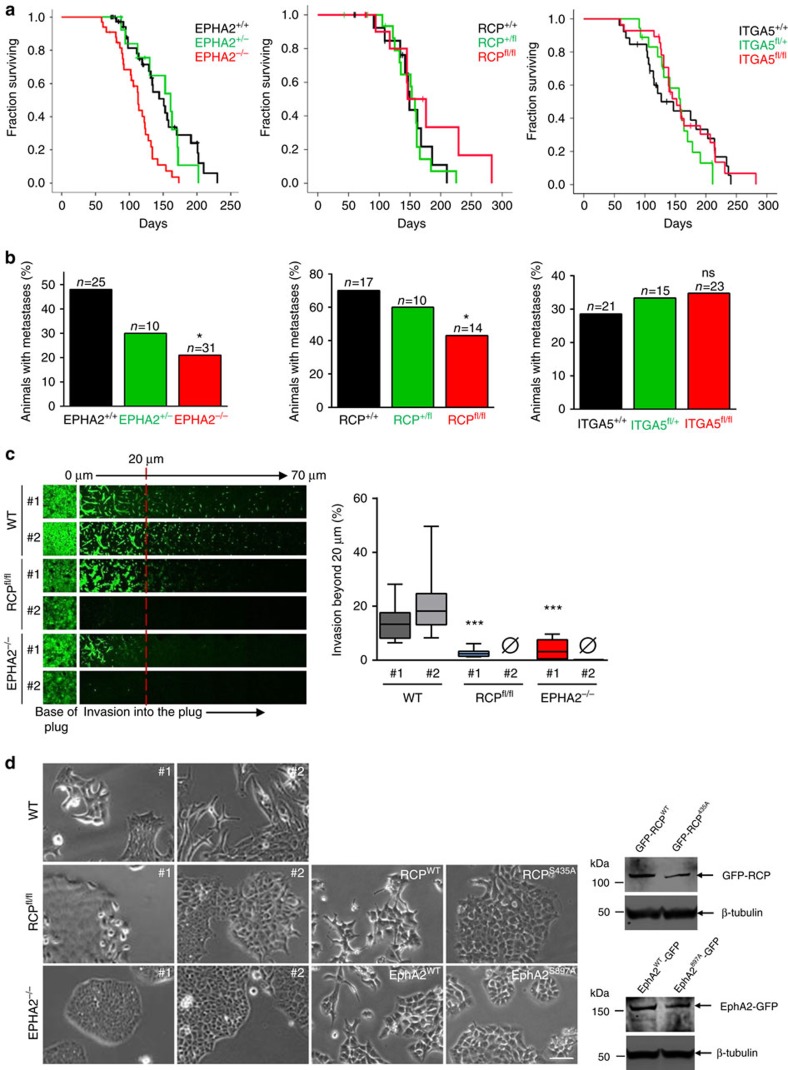
Knockout of EphA2 or RCP reduces invasion and metastasis in pancreatic cancer *in vivo*. (**a**) KPC (Pdx1-Cre:Kras^G12D/+^:p53^R172H/+^) mice were crossed with EPHA2^+/−^ or EPHA2^−/−^ animals or those with floxed RCP (RCP^+/fl^ or RCP^fl/fl^) or floxed α5 integrin (ITGA5^+/fl^ or ITGA5^fl/fl^) alleles. Survival time (which is dictated by the size of the primary tumour) is represented by a Kaplan-Meier curve. Censored mice (indicated by ticks) succumbed to complications other than PDAC. (**b**) The proportion of animals with detectable metastases to liver, lung and other tissues was assessed by gross pathology and confirmed by histology. The number of animals in each category is indicated above the bars. *P<0.05; Comparison is between EPHA2^+/+^ and EPHA2^−/−^, or between RCP^+/+^ and RCP^fl/fl^; Chi-squared test, one-tailed. ns=not significantly different from ITGA5^+/+^ control. (**c**,**d**) Primary mouse cell lines were derived from PDACs harvested from KPC (Pdx1-Cre, Kras^G12D/+^, p53^R172H/+^), KPC:EPHA2^−/−^ and KPC:RCP^fl/fl^ mice. At least three cell lines were derived per condition and the knockout of EphA2 and RCP was confirmed by immunoblotting (see Supplementary Fig. 3c). PDAC cells (two cell lines per condition) were plated onto the underside of transwells containing Matrigel plugs enriched with fibronectin (25 μg ml^−1^). Cells were allowed to migrate into the plugs towards a gradient of HGF and serum for 72 h, and then visualized by Calcein-AM followed by confocal microscopy. Optical sections were taken every 10 μm and consecutive images are displayed as a series running from left to right (**c**; left panels). Cell invasion beyond 20 μm (to the right of the red dashed line) was quantified and expressed as a % of the total quantity of fluorescent cells in the plug (**c**; right panel). Data are represented as box and whiskers plots (whiskers: 10–90 percentile). ***P<0.001; one-way ANOVA (Kruskal-Wallis test, Dunn's Multiple Comparison Test). Data are from three independent experiments. The EPHA2 #1 knockout PDAC line was stably transfected with EphA2-GFP or EphA2^897A^-GFP, and the RCP #2 knockout line was stably transfected with GFP-RCP or GFP-RCP^435A^ as indicated, and the expression of these GFP-tagged proteins was determined by western blotting. The propensity of these PDAC cells to form tight or scattered colonies was determined using phase contrast microscopy (**d**). Bar, 100 μm. Lower magnification images of these fields are displayed in Supplementary Fig. 7d.

Having described the signalling and trafficking events linking RCP to EphA2 function in cell:cell repulsion we wished to determine whether these two proteins contributed to cancer-relevant processes in an appropriate *in vivo* context. To do this we compared the consequences of genetic disruption of RCP and EPHA2 genes in the ‘KPC' mouse model of PDAC (ref. 26). EPHA2^−/−^ mice are viable and have no overt developmental abnormalities. A conditional RCP knockout mouse was generated that contains a Neomycin cassette flanked by LoxP sites following exon 2 of RCP (Supplementary Fig. 7a). Western blots confirmed that RCP expression was abrogated following infection of embryonic fibroblasts from RCP^fl/fl^ mice with a Cre-expressing adenovirus (Supplementary Fig. 7b). In the KPC model, the size of the primary tumour (not the metastases) dictates survival. Knockout of RCP had no significant effect on the survival of KPC mice, while EphA2 knockout reduced survival, indicating that initiation and growth of primary PDACs are not dependent on expression of either protein (Fig. 7a). However, knockout of either EPHA2 or RCP significantly reduced the incidence of metastases in KPC mice (Fig. 7b).

RCP is involved in trafficking of receptors other than EphA2, and α5β1 integrin is an RCP cargo that controls cell migration and invasion^10^,^27^,^28^. We, therefore, used α5 integrin (ITGA5) floxed mice to determine whether this RCP cargo was also involved in the growth and metastasis of PDAC. Interestingly, knockout of ITGA5 did not influence growth or metastasis of PDAC (Fig. 7a,b), indicating that RCP's ability to traffic EphA2 is more closely associated with tumour dissemination than is the trafficking of its integrin cargo in tumour cells.

To determine whether reduced metastasis was owing to a requirement for EphA2 and RCP in invasive/migratory behaviour of PDAC cells, we isolated a number of cell lines from both control and knockout primary tumours. Western blotting confirmed that cells from control tumours expressed both EphA2 and RCP protein, and that genetic disruption of the EPHA2 and RCP genes led to disappearance of their protein products (Supplementary Fig. 7c). Cells from control tumours were highly invasive, and this was significantly reduced by knockout of EPHA2 or RCP, consistent with a tumour cell-autonomous role for these proteins in invasive behaviour (Fig. 7c). Cells derived from KPC tumours typically form loose colonies with many migratory cells at the colonies' edges (Fig. 7d; Supplementary Fig. 7d). By contrast, cells from RCP and EPHA2 knockout tumours formed closely-knit colonies with a defined edge and little indication that cells were able to migrate away from the colonies (Fig. 7d; Supplementary Fig. 7d)). Importantly, expression of EphA2-GFP or GFP-RCP restored the scattered morphology of EPHA2^−/−^ and RCP^−/−^ PDAC cells respectively, whereas EphA2-GFP^897A^ and GFP-RCP^435A^ were ineffective in this regard (Fig. 7d). Furthermore, re-expression of GFP-RCP was able to rescue the scattered phenotype of PDAC cells despite the somewhat reduced levels of EphA2 that were observable following RCP knockout (Fig. 7d; Supplementary Fig. 7c ). These data demonstrate that both RCP and EphA2 are required for efficient invasion and metastasis of pancreatic cancer, and indicate the likelihood that both of these proteins and their phosphorylation-dependent trafficking contribute to cell repulsion and dissemination of PDAC.

## Discussion

LMTKs clearly control endocytic traffic. LMTK1 regulates Rab11 recruitment to neuronal endosomes^29^ and LMTK3 knockout leads to locomotor defects in mice which are associated with impaired endocytic trafficking^30^. Moreover, LMTK2 controls receptor transfer between early and recycling endosomes^31^, which is the point at which we find RCP/Rab14 to influence EphA2 trafficking. Despite these advances, the mechanism through which LMTKs control endosomal function has hitherto been unclear. We show that LMTK3 controls endosomal trafficking by phosphorylating RCP to alter its preference for GTPase binding. Previous yeast-2-hybrid and co-immunoprecipitation studies indicate that Rab11 and Rab14 likely compete for binding to RCP's well-characterized Rab-binding domain^21^, but recent work indicates a more complicated situation. Mutation of residues in RCP's central region (which is distant from the Rab-binding domain) selectively opposes Rab14 binding^22^. Consistently, our data indicate that phosphorylation of a residue in a similar region promotes Rab14 (but not Rab11) association which, in turn, promotes RCP's association with EphA2. Thus although our observations provide evidence for a molecular mechanism through which growth factor signalling can switch the Rab GTPase and cargo preference of a Rab11 effector to favour cell repulsion, further work will be necessary to establish the allosteric and intramolecular events through which LMTK3 controls GTPase and cargo association with RCP.

LMTK3 is a determinant in breast and gastric cancer aggressiveness^32^,^33^. Various molecular explanations have been offered for this, including the possibility that LMTK3 influences oestrogen receptor signalling^32^, and the kinase has been reported to exert post-transcriptional control over α5β1 integrin levels in breast cancer cells^34^. Integrin recycling is controlled by RCP (ref. 27), and it is possible that in breast cancer cells LMTK3 may oppose α5β1 degradation by phosphorylating RCP. However, we do not detect alteration in α5β1 levels following knockdown of LMTK3, nor do we find that this integrin contributes to HGF-driven cell:cell repulsion or to metastasis of PDAC *in vivo*. Thus although it is possible that LMTK3 may control integrin trafficking to influence invasiveness in breast cancer, our results indicate that this kinase contributes to PDAC dissemination and metastasis primarily via phosphorylation of RCP and regulation of EphA2 trafficking to promote cell:cell repulsion. We have recently shown that knockout of RCP in a mouse model of breast cancer does not oppose metastasis^35^, indicating that the contribution made by RCP trafficking to metastasis depends on the cancer type. Furthermore, cell repulsion not only drives invasiveness if it occurs in a homotypic fashion, but also can oppose invasiveness if heterotypic repulsion events involving the tumour stroma are able to dominate^36^. Further work will be necessary to fully elucidate how Eph trafficking drives cell dissemination in various cancer types.

It would seem natural to propose that endosomal recycling might contribute to functional Eph signalling by concentrating the receptor within cell:cell contacts where it encounters its ligand on neighbouring cells. However, our data are not consistent with a mechanism such as this. Firstly, knockdown of ephrin-A1 (the cognate EphA2 ligand expressed by H1299 cells) or mutation of EphA2's Tyr^588^ (which opposes ephrin ligand-induced EphA2 trafficking (Supplementary Fig. 5c)) does not influence EphA2-dependent cell scattering. Secondly, we have found that phosphorylation of EphA2 at Ser^897^—an Ephrin ligand-independent event—is key to EphA2's trafficking through the RCP/Rab14 pathway and its ability to drive cell:cell repulsion. So how does EphA2 endosomal trafficking contribute to HGF-driven cell:cell repulsion? Under basal conditions, EphA2 is returned directly to the plasma membrane (and frequently we observe this to occur near cell:cell contacts) and the receptor is not transported to perinuclear RCP-positive endosomes. Surprisingly, our data indicate that this rapid constitutive internalization and recycling does not initiate functional EphA2 signalling, but it may help to maintain a reservoir of EphA2 at cell:cell contacts in preparation for signals that trigger cell:cell repulsion. Indeed, a recent report has demonstrated that constitutive transit of EphA2 through Rab11-positive endosomes brings the receptor into contact with tyrosine phosphatase PTP1B to ensure that low background levels of tyrosine phosphorylated EphA2 are maintained in the absence of ligand-engagement^37^. This study indicated that engagement of EphA2 with ephrin-A1 then diverts the receptor from the Rab11 pathway and routes it to Rab7-positive late endosomes. Our data indicate that a significant fraction of EphA2 is diverted from a constitutive recycling pathway by activated HGF-signalling, and that this requires a signalling event (phosphorylation of Ser^897^) that is not associated with EphA2-ephrin engagement at the plasma membrane. Moreover, the receptor's destination following activation of HGF-signalling is not late endosomal, but a subset of RCP/Rab14-positive endosomes which are located very close to the nucleus. HGF signalling achieves this by concomitant activation of two parallel phosphorylation cascades. HGF activates Akt to phosphorylate EphA2 on Ser^897^ and, in parallel with this, HGF signalling promotes LMTK3-mediated phosphorylation of RCP on Ser^435^ to favour RCP/Rab14 association and both of these events are necessary to divert EphA2 through a slower recycling pathway and promote cytoskeletal responses which enable cells to move apart.

**Figure 8:**
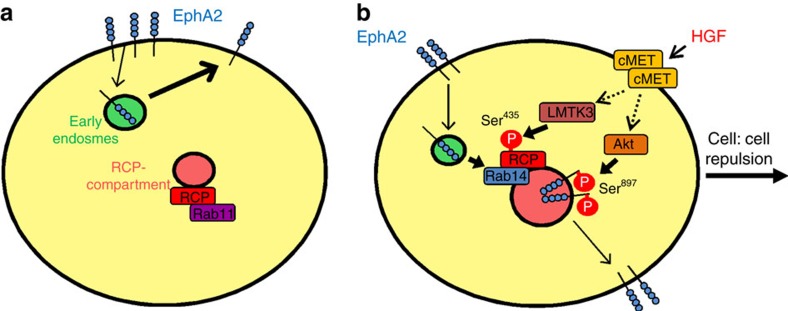
Control of EphA2 trafficking during cell scattering. (**a**) EphA2 is constitutively internalized and returned to the plasma membrane without transit through RCP-positive endosomes, and RCP is associated primarily with Rab11. (**b**) Addition of HGF triggers LMTK3-dependent phosphorylation of RCP at Ser^435^ which favours RCP's association with Rab14. In parallel with this, HGF promotes Akt-mediated phosphorylation of EphA2 at Ser^897^. Both of these phosphorylation events are necessary to promote diversion of EphA2 from a rapid recycling pathway to a slower one which transits through RCP/Rab14-positive endosomes, and to enable EphA2-dependent cell:cell repulsion and scattering.

It is interesting to speculate how diversion of EphA2 from a rapid recycling pathway and into one that increases its dwell-time within the cell might activate cell:cell repulsion. MT1-MMP-mediated cleavage of EphA2 has been shown to promote cell repulsion by permitting internalization of the receptor leading to its accumulation within perinuclear endosomes to activate Rho signalling^20^. Thus, signals downstream of EphA2 that promote repulsion are likely to be propagated during transit through the endosomal system at a point that is distant from the plasma membrane. Many receptors activate their effector signalling pathways following internalization when they are present in endosomes. Most pertinently, internalized EphA2 has been shown to retain the capacity to signal to Rho subfamily GTPases, which are key to the implementation of cell:cell repulsion, when the receptor is moving through the endosomal pathway^38^. Thus, our data suggest a mechanism in which cell:cell repulsion is initiated by phosphorylation of serines on RCP and EphA2 which conspire to divert EphA2 from a rapid recycling pathway to a slower one involving transit through perinuclear Rab14-postive endosomes, and it is during this journey that the receptor engages signalling pathways that drive repulsion (Fig. 8).

Our results have identified a new pathway mechanistically linking pro-invasive growth factor signalling to cell:cell repulsion. This is mediated by phosphorylation of a Rab effector to alter its Rab GTPase preference which, in turn, influences Eph receptor trafficking (Fig. 8). In parallel with this we have shown that both RCP and EphA2 are required for efficient invasion and metastasis in an *in vivo* model of pancreatic cancer, and to maintain a cell:cell repulsion phenotype in pancreatic tumour cells *ex vivo*. Thus by providing molecular insight into how pro-invasive signalling initiates cell:cell repulsion, and by showing that components of this pathway contribute to metastasis *in vivo*, we have made an important step into designing therapies that might interfere with these processes.

## Methods

### Cell culture and transfection

A2780 cells lines were kindly donated by Gordon Mills, MD Anderson Cancer Centre, Texas, USA. PC3 cells were kindly provided by Kate Nobes, University of Bristol, UK. The expression of Ephs and ephrins and the contribution made by these to cell:cell repulsion in these particular cells has been characterized^19^. H1299 cells were obtained from ATCC. The genetic identity of all these cell lines has been confirmed at the CRUK Beatson Institute for Cancer Research. Cell lines were cultured at 37 °C and 10% CO_2_ in a humidified incubator. A2780 and PC3 cells were cultured in RMPI-1640, while H1299 cells and in-house PDAC cells were cultured in DMEM. All media were supplemented with 10% fetal calf serum, 2 mM L-glutamine, 100 IU ml^−1^ penicillin, 100 μg ml^−1^ streptomycin and 250 μg ml^−1^ fungizone. Cells were transfected with expression vectors and siRNAs using the Amaxa Nucleofector system; A2780 cells (kit T), H1299 and PC3 cells (kit V).

### Antibodies and immunoprecipitation

For western blotting (WB) and immunofluorescence (IF), antibodies were from the following sources: mouse anti-EphA2 (Millipore, catalogue number 05-480, dilution 1:1,000 WB, 1:200 IF), rabbit anti-FIP2 (Proteintech; catalogue number, 10837-1-AP; dilution 1:500 WB), rabbit anti-FIP3 (a generous gift from Mary McCaffrey; dilution 1:500 WB), rabbit anti-phosphoSer^435^RCP (was raised by immunizing rabbits to KLH-conjugated RCP^427-439^(pSer^435^) followed by affinity purification with the same peptide; Eurogentech; dilution 1:5,000 WB, 1:200 IF), rabbit anti-Rab11 (Invitrogen; catalogue number, 71-5300; dilution 1:1,000 WB), rabbit anti-Rab14 (Novus Biologicals; catalogue number, NBPI 84720; dilution, 1:500 WB), rabbit anti-RCP (raised against in-house purified RCP^379-649^; Eurogentech; dilution 1:10,000 WB, 1:3,000 IF), mouse anti-α5 integrin (BD Transduction Labs; catalogue number, 610633; dilution 1:1,000 WB), rabbit anti-EphA2^pS897^ (Cell Signaling Technology; catalogue number, 6347; dilution 1:1,000 WB, 1:200 IF) and rabbit anti-EphA2^pY588^ (Cell Signaling Technology; catalogue number, 12677; dilution 1:1,000 WB).

Mouse anti-EphA2 (Millipore; catalogue number 05-480) and mouse anti-GFP (Abcam; catalogue number ab1218) were used for immunoprecipitation. Mouse anti-GFP or anti-EphA2 was coupled to magnetic beads conjugated to anti-mouse IgG (Invitrogen; Dynabeads Sheep anti-mouse IgG; catalogue number 11031). Cell lysates were prepared in a lysis buffer containing 200 mM NaCl, 75 mM Tris-HCl pH 7, 15 mM NaF, 1.5 mM Na_3_VO_4_, 7.5 mM EDTA, 7.5 mM EGTA, 0.15% (v/v) Tween-20, 50 μg ml^−1^ leupeptin, 50 μg ml^−1^ aprotinin and 1 mM 4-(2-aminoethyl)-benzenesulfonyl fluoride. Lysates were passed three times through a 27-gauge needle and clarified by centrifugation at 10,000*g* for 10 min at 4 °C. Lysates were added to the beads and rotated for 2 h at 4 °C. The beads were washed three times in Tween-20-containing buffer, and then analysed for protein content either by MALDI-TOF mass spectrometry or by immunoblotting.

### Internalization assay and capture-ELISA

Cell surface proteins were labelled with membrane impermeable sulfo-NHS-SS-Biotin (0.13 mg ml^−1^) in PBS for 30 min at 4 °C. To allow internalization, cells were incubated at 37 °C for the appropriate times in the presence and absence of HGF (10 ng ml^−1^) or primaquine (0.6 mM). To remove biotin from proteins remaining at the cell surface, cells were incubated with sodium mercaptoethanesulphonate (MesNa; 20 mM) for 60 min at 4 °C. Excess MesNa was then quenched by addition of iodoacetamide (20 mM) for a further 10 min at 4 °C. The plates were washed and lysed in a buffer containing 1.5% Triton X-100 and the levels of biotinylated-EphA2 were determined by capture-ELISA.

### Live cell imaging and fluorescently-tagged EphA2

To image EphA2 trafficking, the full-length sequence of human EphA2 was inserted into pEGFP-N1 vector using the Sal1 (3') and Xho1 (5') sites. mCherry-RCP constructs were generated by subcloning the open reading frame of RCP into pmCherry-C1 (ref. 28). Transfected cells were seeded onto glass-bottomed plates and, 24 h later, were viewed using an Olympus FV-1000 microscope. Cells were incubated in the presence or absence of HGF (10 μg ml^−1^) for 15 min and then 2 min movies (6 s frame interval) were collected. Co-localization analyses were performed using the ImageJ Co-localization Threshold plugin. Briefly, this plugin automatically determined the threshold intensity levels using the Costes method^39^ and subsequently generated the fraction of co-localizing pixels above the threshold in both channels. Co-localization values are presented as the mean values of co-localizing pixels at least three experiments, comprising at least ten randomly chosen cells for each experiment. The same approach was used for quantification of co-localization between endogenous EphA2^pS897^ and GFP-RCP in fixed cells.

### Airyscan imaging

Single plane images including the perinuclear region were acquired using a Zeiss LSM 880 Airyscan confocal microscope. Images were processed and subject to deconvolution using Zen Black Zeiss software.

### Cell:cell repulsion and scattering assays

To measure cell:cell repulsion, six-well glass-bottomed plates were coated in 150 μl Matrigel in 300 μl 0.5% Serum RPMI-1640 at room temperature for 1 h. PC3 cells (200 cells per well) were seeded onto the Matrigel-coated surface and incubated at 37 °C for 24 h. Cells were incubated in serum-reduced medium (0.5% serum) for a further 24 h, and then HGF (10 ng ml^−1^) was added to elicit cell migration. Cell migration was visualized on a Nikon timelapse microscope. Images were collected every 5 min from eight different regions in each well for 20 h. The time of cell-cell contact during collision was measured for any collision in which only two cells were involved and both cells were migrating towards each other. Cell contact was assessed by close inspection of the phase contrast frames from the movies, and cells were classified as ‘contacting' if the envelope of their plasma membranes were closely apposed. Thus this classification would exclude cells that were connected by fine membranous contacts or retraction fibres that might remain following a productive repulsion event.

To measure HGF-induced scattering, H1299 cells (2,000 cells per well) were seeded onto six-well plates for 48 h, during which time the cells formed small colonies. Cell movement was then visualized on a Nikon timelapse microscope in the presence and absence of HGF (10 ng ml^−1^). Images were collected every 5 min from eight different regions in each well. To track scattering, ImageJ manual tracking and chemotaxis plugins were used. Every cell that was in a colony of 4-10 cells and did not divide was tracked for 6 h.

### KPC mouse model of PDAC

KPC (Pdx1-Cre, Kras^G12D/+^, p53^R172H/+^) mice are as described in ref. 40. EphA2^−/−^ mice were obtained from Jackson laboratories, and RCP^fl/fl^ mice were generated in-house by the Beatson transgenic production service. PCR was used to check the genotype of the mice (Transnetyx Inc). ITG5A floxed mice were a generous gift from Richard Hynes (MIT, USA) and are as described in ref. 41. Mice were monitored daily and kept in conventional animal facilities. All experiments were performed in compliance with UK Home Office guidelines. Tumourigenesis was assessed by gross pathology and confirmed by histology.

### Data availability

The data and reagents that support the findings of this study are available from the corresponding author on request.

## Additional information

**How to cite this article:** Gundry, C. *et al.* Phosphorylation of Rab-coupling protein by LMTK3 controls Rab14-dependent EphA2 trafficking to promote cell:cell repulsion. *Nat. Commun.* **8,** 14646 doi: 10.1038/ncomms14646 (2017).

**Publisher's note:** Springer Nature remains neutral with regard to jurisdictional claims in published maps and institutional affiliations.

## Supplementary Material

Supplementary Movie 1 Cell:cell repulsion of PC3 cells. PC3 cells were transfected with non-targeting siRNA control oligonucleotides. Cells were sparsely seeded onto glass-bottomed wells coated with 50% Matrigel. Cells were serum-starved for 24 hr and then treated with HGF (10 ng ml-1) to initiate cell migration. Cell movement was recorded using time-lapse video microscopy with frames being collected every 5 min.

Supplementary Movie 2 Knockdown of RCP opposes efficient cell:cell repulsion. PC3 cells were transfected with siRNAs targeting RCP and seeded onto glass-bottomed wells and serum-starved as for supplementary movie 1. Cells were treated with HGF (10 ng ml-1) to initiate cell migration. Cell movement was recorded using time-lapse video microscopy with frames being collected every 5 min.

Supplementary Movie 3 Trafficking of EphA2 and RCP. H1299 cells were transfected with GFP-EphA2 in combination with mCherry-RCP and plated onto glass-bottomed dishes. 24 hr following transfection, confocal time-lapse movies were collected with 2 sec frame intervals over a 2 min period.

Supplementary Movie 4 HGF promotes trafficking of EphA2 to the RCP compartment. H1299 cells were transfected with GFP-EphA2 in combination with either mCherry-RCP and plated onto glass-bottomed dishes. 24 hr following transfection, HGF (10 ng ml-1) was added and, 30 min following HGF addition, confocal time-lapse movies were collected with 2 sec frame intervals over a 2 min period.

Supplementary Movie 5 Trafficking of EphA2 and Rab14. H1299 cells were transfected with GFP-EphA2 in combination with mCherry-Rab14 and plated onto glass-bottomed dishes. 24 hr following transfection, confocal time-lapse movies were collected with 2 sec frame intervals over a 2 min period.

Supplementary Movie 6 HGF promotes trafficking of EphA2 to the RCP compartment. H1299 cells were transfected with GFP-EphA2 in combination with either mCherry-Rab14 and plated onto glass-bottomed dishes. 24 hr following transfection, HGF (10 ng ml-1) was added and, 30 min following HGF addition, confocal time.

Supplementary Information Supplementary Figures

Peer Review File

## Figures and Tables

**Table 1:** Proteomic analysis of GFP-RCP immunoprecipitates.

**Protein**	**Accession number**	**IP:GFP**		**IP:GFP-RCP**	
		**Number of peptides (unique)**	**Percentage covered (%)**	**Number of peptides (unique)**	**Percentage covered (%)**
Ubiquitin	P62988	—	—	4 (3)	50
Rab11B	Q15907	—	—	53 (8)	40
Heat shock cognate 71 kDa	P11142	1 (1)	3	43 (16)	32
Rab6A	P20340	—	—	18 (5)	25
α-actinin 4	O43707	—	—	32 (16)	23
Rab11-FIP1 (RCP)	Q6WKZ4	—	—	60 (17)	16
Clathrin heavy chain 1	Q00610	—	—	27 (15)	13
Rab11-FIP5	Q9BXF6	—	—	11 (5)	13
Rab14	P61106	—	—	6 (2)	13
5'-nucleotidase	P21589	1 (1)	2	9 (5)	13
Myoferlin	Q9NZM1	—	—	34 (20)	12
Aminopeptidase N	P15144	—	—	21 (9)	12
Ephrin type-A receptor 2 (EphA2)	P29317	—	—	18 (9)	12
Peroxisomal multifunctional enzyme type 2	P51659	—	—	11 (5)	10
Desmoglein 2	Q14126	—	—	8 (6)	10
Myeloid-associated differentiation marker	Q96S97	—	—	8 (2)	10
Heat shock 70 kDa protein 1	P08107	—	—	3 (3)	10
β1 integrin	P05556	1 (1)	1	17 (5)	9
EGFR	P00533	—	—	17 (7)	8
Neurabin 2	Q96SB3	—	—	7 (6)	8
4F2 cell-surface antigen heavy chain	P08195	—	—	3 (3)	8

Immunoprecipitates generated in Fig. 1a were analysed by MALDI-TOF mass spectrometry. Proteins that were significantly more abundant in GFP-RCP (IP:GFP-RCP) than GFP (IP:GFP) immunoprecipitates are listed. The number of peptides identified by mass spectrometry and the percentage of these proteins covered by the identified peptides are indicated.
