# Supplementary material for: Phosphorylation of Rab-coupling protein by LMTK3 controls Rab14-dependent EphA2 trafficking to promote cell:cell repulsion
Source: Nat Commun. 2017 Mar 15;8:14646. doi: 10.1038/ncomms14646 (PMC5355957; doi:10.1038/ncomms14646)
Supplement: Supplementary Information — Supplementary Figures [file ncomms14646-s7.pdf]

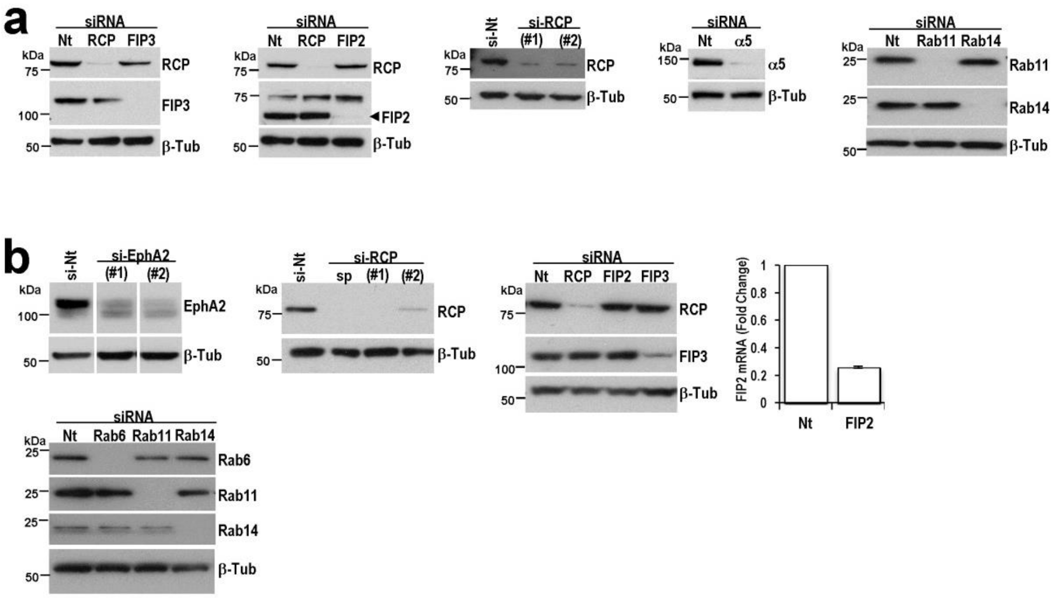

Supplementary Figure 1. Confirmation of siRNA in PC3 and H1299 cells

PC3 (a) and H1299 (b) cells were transfected with siRNA oligonucleotides targeting RCP (SMARTPool (RCP) or two individual oligos (RCP#1 & RCP#2), EphA2 (two individual oligos (EphA2#1 & EphA2#2), FIP2, FIP3, α5 integrin (α5), Rab14, Rab11, Rab6 or a non-targeting control (si-Nt). 24 hr following transfection, cells were lysed and the levels of the indicated proteins determined by immunoblotting or by qPCR (for FIP2 in H1299 cells).

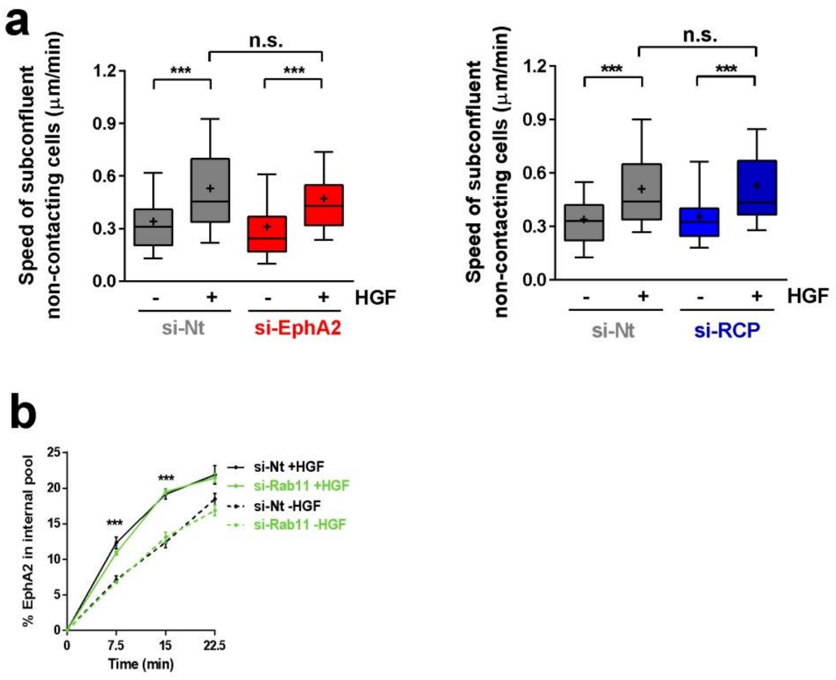

Supplementary Figure 2. RCP and EphA2 are not required for HGF-driven migration of non-contacting H1299 cells, and knockdown of Rab11 does not influence HGF-driven cellular accumulation of EphA2

(a) H1299 cells were transfected with siRNAs targeting RCP (si-RCP), EphA2 (si-EphA2) or a non-targeting control (si-Nt). Transfected cells were seeded sparsely for sufficient time for them to attach, but not to divide and form colonies. Cells were then treated with HGF (10 ng ml^{-1}) and cell migration was recorded using time-lapse video microscopy. Cell migration speed was quantified using ImageJ manual tracking and chemotaxis plugin. Cells were excluded from this analysis if they contacted another cell. Data are represented as box and whiskers plots (whiskers: 10 - 90 percentile, + represents the mean). *** $p<0.001$; Mann-Whitney test. Data are from 3 independent experiments with >50 cells being tracked per condition.

(b) H1299 cells were transfected with siRNAs targeting Rab11a and Rab11b (si-Rab11) or a non-targeting control (si-Nt) and plated onto 10 cm plastic dishes. Cells were surface-labelled with NHS-S-S-Biotin (0.13 mg ml^{-1}) at 4ºC and internalization allowed to proceed at 37ºC for the indicated times with or without HGF (10 ng ml^{-1}). Biotin remaining at the cell surface was removed by exposure to MesNa at 4°C and the quantity of biotinylated receptors within the cells determined by capture-ELISA using microtitre wells coated with monoclonal antibodies recognising EphA2. Values are mean ± SEM, n=4 biological replicates from 2 independent experiments, *** $p<0.001$ (si-Rab11 + HGF versus si-Nt – HGF); Two-way ANOVA, Bonferroni post-test.

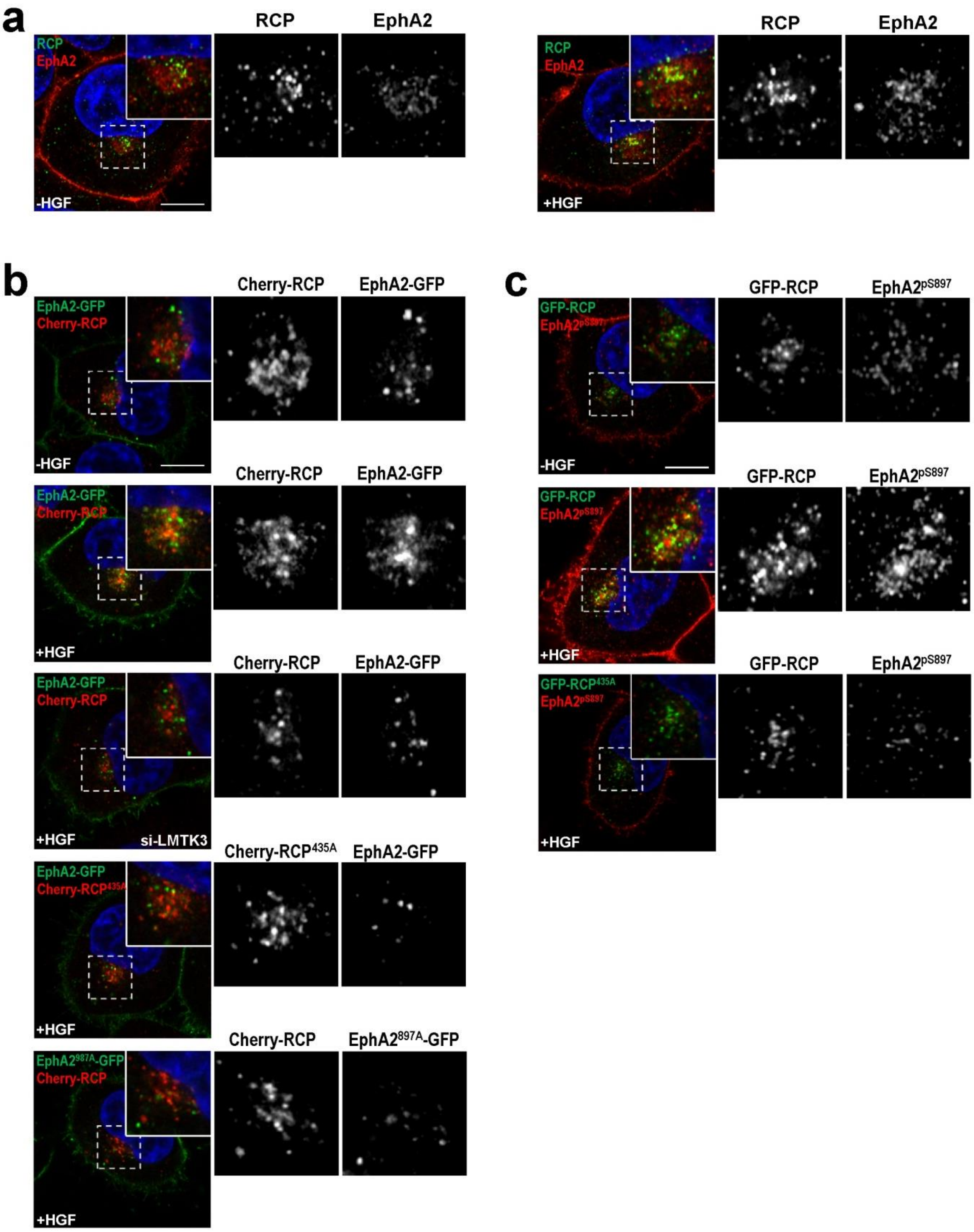

Supplementary Figure 3. High resolution imaging of EphA2 and RCP localization.

(a) Untransfected H1299 cells were incubated in the presence or absence of HGF (10 ng ml^{-1}) and then fixed in 4% paraformaldehyde. Endogenous RCP, EphA2 and nuclei were visualised by immunofluorescence followed by high resolution imaging using an Airy-scan microscope. Bar, 10 µm. These images are whole cell fluorescence micrographs and single channel fluorescence images corresponding to the merged and cropped images displayed in the right panels of Figure 3e.

(b) H1299 cells were transfected with EphA2-GFP or EphA2^{897A}-GFP in combination with mCherry-RCP or mCherry-RCP^{435A}, in the presence or absence of siRNAs targeting LMTK3 (si-LMTK3) as indicated. Cells were incubated in the presence or absence of HGF (10 ng ml^{-1}), fixed in 4% paraformaldehyde and EphA2-GFP and mCherry-RCPs visualised using a high resolution Airy-scan microscope. Bar, 10 μm.

(c) H1299 cells were transfected with GFP-RCP or GFP-RCP^{435A}. Transfected cells were incubated in the presence or absence of HGF (10 ng ml^{-1}) and then fixed in 4% paraformaldehyde. Endogenous phospho-Ser^{897}-EphA2 (EphA2^{pS897}) was stained with a phosphospecific antibody and visualised with respect to GFP-RCPs using an Airy-scan microscope. Bar, 10 μm.

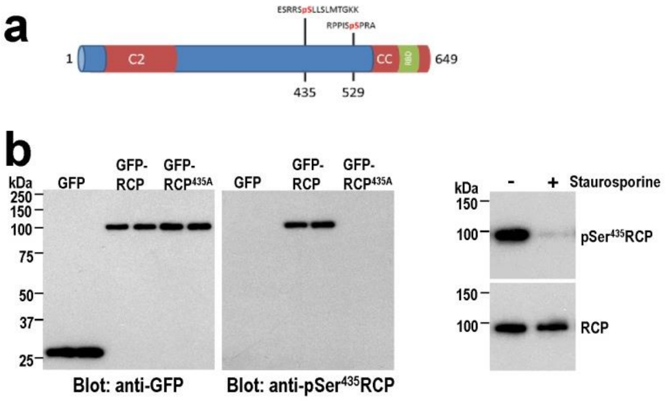

Supplementary Figure 4. Validation of anti-phosphoSer^{435}RCP

(a) Schematic representation of the domain structure of RCP indicating two phosphopeptides identified by mass spectrometry.

(b) A2780 cells were transfected with GFP, GFP-RCP or GFP-RCP^{435A} and plated onto 15 cm plastic dishes. 48 hr following transfection, cells were lysed and analysed by immunoblotting using an antibody raised in rabbits against a synthetic peptide corresponding to RCP$^{427\text{-}439}$(pSer^{435}), anti-GFP and anti-RCP as indicated. In the right panel, cells were treated with staurosporine or vehicle control 30 min prior to lysis.

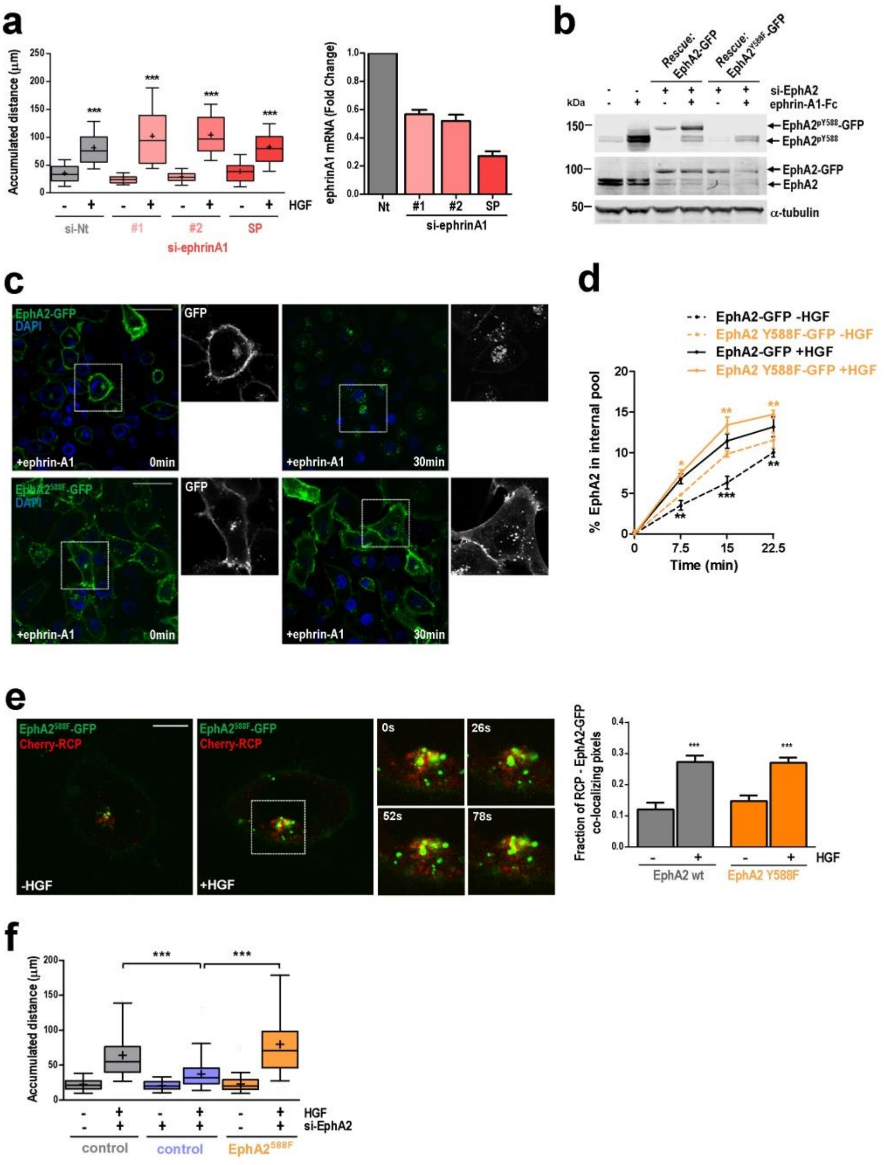

Supplementary Figure 5. Ligand-dependent signalling of EphA2

(a) H1299 cells were transfected with a siRNAs targeting ephrin A1 (si-ephrinA1; a SMARTPool (SP) or two individual siRNA oligonucleotides #1 & #2) or a non-targeting control (si-Nt). Cell scattering in the presence and absence of HGF was then determined and quantified as for Figure 2d-e. Data are represented as box and whiskers plots (whiskers: 10 - 90 percentile, + represents the mean). Data are from 3 independent experiments. The right panel displays the effectiveness of the ephrin A1 knockdown as determined by qPCR.

(b) H1299 cells were transfected with a non-targeting siRNA or with an siRNA targeting EphA2 (si-EphA2) in combination with siRNA-resistant forms of EphA2-GFP or EphA2^{588F}-GFP. Cells were treated with recombinant ephrinA1 (ephrinA1-Fc) for 10 min, and the expression levels and tyrosine phosphorylation of the siRNA-resistant forms of EphA2-GFP

and EphA2^{588F}-GFP were determined in EphA2 knockdown cells by Western blotting with antibodies recognising EphA2 or pTyr^{588}-EphA2.

(c) H1299 cells expressing EphA2-GFP or EphA2^{588F}-GFP were treated with recombinant ephrin-A1 for 30 min or were left untreated. Cells were fixed and the distribution of the GFP-tagged Ephs (green signal) determined using confocal microscopy. The cells were counterstained with DAPI (blue) to visualise nuclei. Bar, 20 μm.

(d) H1299 cells were transfected with a siRNA targeting EphA2 in combination with siRNA-resistant forms of EphA2-GFP or EphA2^{588F}-GFP. Internalisation of EphA2-GFPs in the presence and absence of HGF was then determined as for Figure 3b, but with the ELISA plate coated with anti-GFP to specifically detect only the GFP-tagged receptor. Values are mean ± SEM n=4 biological replicates from 2 independent experiments. Black asterisks *** $p<0.001$, ** $p<0.01$ (EphA2^{wt}-GFP + HGF versus EphA2^{wt}-GFP - HGF); Orange asterisks ** $p<0.01$, * $p<0.05$ (EphA2^{588F}-GFP + HGF versus EphA2^{588F}-GFP - HGF); two-way ANOVA, Bonferroni post-test.

(e) H1299 cells were transfected with EphA2-GFP or EphA2^{588F}-GFP in combination with mCherry-RCP. Trafficking of EphA2-GFP and mCherry-RCPs in the presence and absence of HGF (added 30 min prior to collecting the movies) was visualised by fluorescence confocal time-lapse microscopy and colocalisation was quantified as for Figure 3e. Stills were extracted from movies at the indicated time points, and these display the region of interest indicated by the white box (only stills from the EphA2^{588F}-GFP movies are displayed). Bar, 10 μm. Fractions of co-localizing pixels are mean ± SEM from 3 separate experiments incorporating >10 cells/condition/experiment. ** $p<0.01$; 1 way ANOVA (Kruskal-Wallis test, Dunn's Multiple Comparison Test).

(f) H1299 cells were transfected with a non-targeting siRNA or with an siRNA targeting EphA2 (si-EphA2) in combination with a control vector (control) or an siRNA-resistant form of EphA2^{588F}-GFP. Cell scattering in the presence and absence of HGF was then determined and quantified as for Figure 6c. Data are represented as box and whiskers plots (whiskers: 10 - 90 percentile, + represents the mean). *** $p<0.001$; 1 way ANOVA (Kruskal-Wallis test, Dunn's Multiple Comparison Test). Data are from 3 independent experiments with >50 cells being tracked per condition.

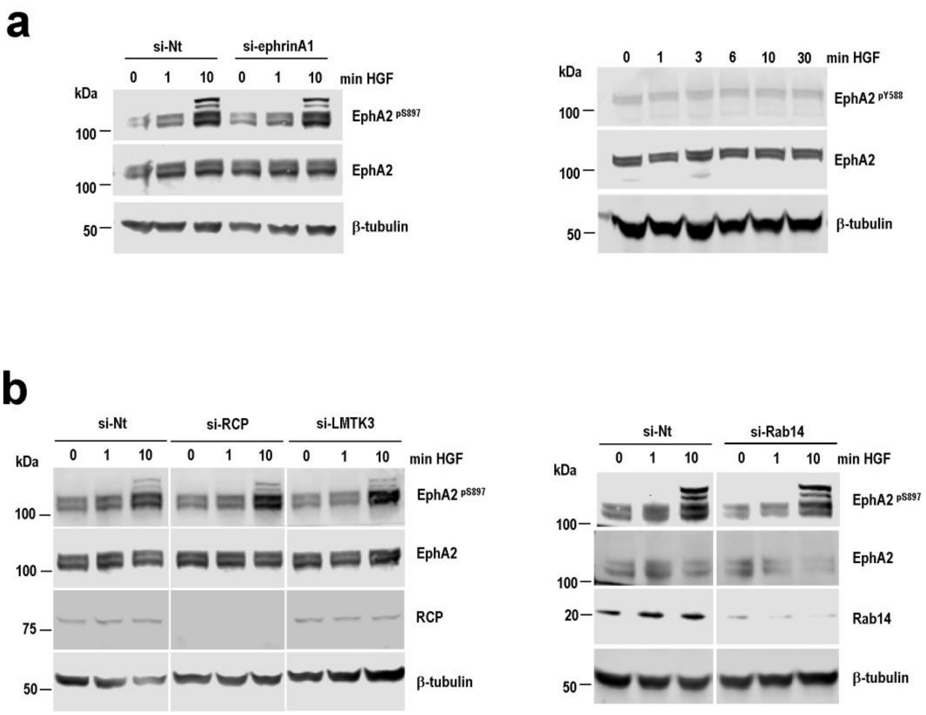

Supplementary Figure 6. Ligand-independent signalling of EphA2

H1299 cells were transfected with siRNAs targeting ephrin-A1 (si-ephrinA1) (a), RCP (si-RCP), Rab14 (si-Rab14), LMTK3 (si-LMTK3) (b), a non-targeting control (si-Nt) or were left untransfected. Cells were treated with HGF (10 ng ml^{-1}) for the indicated times and then lysed. Levels of phosphor-Ser^{897}-EphA2 ($EphA2^{pS897}$), phospho-Tyr^{588}-EphA2 ($EphA2^{pY588}$), EphA2, RCP and Rab14 were determined by Western blotting as indicated.

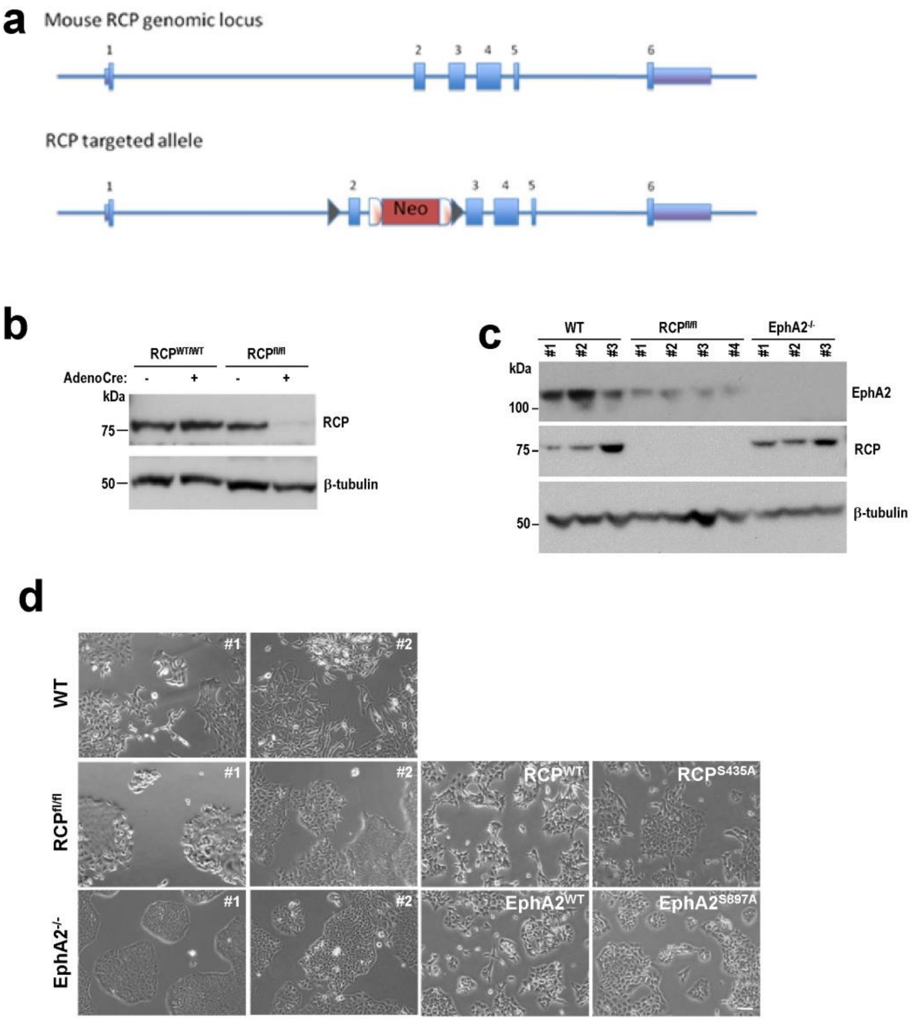

Supplementary Figure 7. Generation and validation of mice with floxed RCP alleles

(a) Schematic representation of the targeting construct used to generate RCP floxed mice. A conditional RCP allele was generated by flanking exon 2 of the RCP gene with LoxP sites in mouse ES cells. Mice were generated by injection of ES cells into blastocysts and implantation of these into pseudopregnant animals. The neomycin cassette was subsequently removed.

(b) Effective RCP gene deletion was confirmed by adenoviral infection of Cre recombinase into MEFs derived from RCP$^{fl/fl}$ animals followed by immunoblotting using in-house antibodies recognising mouse RCP.

(c) Primary mouse cell lines were derived from PDACs harvested from KPC (Pdx1-Cre, Kras$^{G12D/+}$, p53$^{R172H/+}$), KPC:EPHA2$^{-/-}$ and KPC:RCP$^{fl/fl}$ mice. At least 3 cell lines were derived per condition and the knockout of EphA2 and RCP was confirmed by immunoblotting.

(d) Primary mouse cell lines were derived from PDACs harvested from KPC (Pdx1-Cre, Kras$^{G12D/+}$, p53$^{R172H/+}$), KPC:EPHA2$^{-/-}$ and KPC:RCP$^{fl/fl}$ mice. The EPHA2 #2 knockout PDAC

line was stably transfected with EphA2-GFP or EphA2^{897A}-GFP, and the RCP #2 knockout line was stably transfected with GFP-RCP or GFP-RCP^{435A} as indicated. The propensity of these cells to form tight or scattered colonies was determined using phase contrast microscopy (d), Bar, 100µm. Higher magnification images of these fields are displayed in Figure 7d

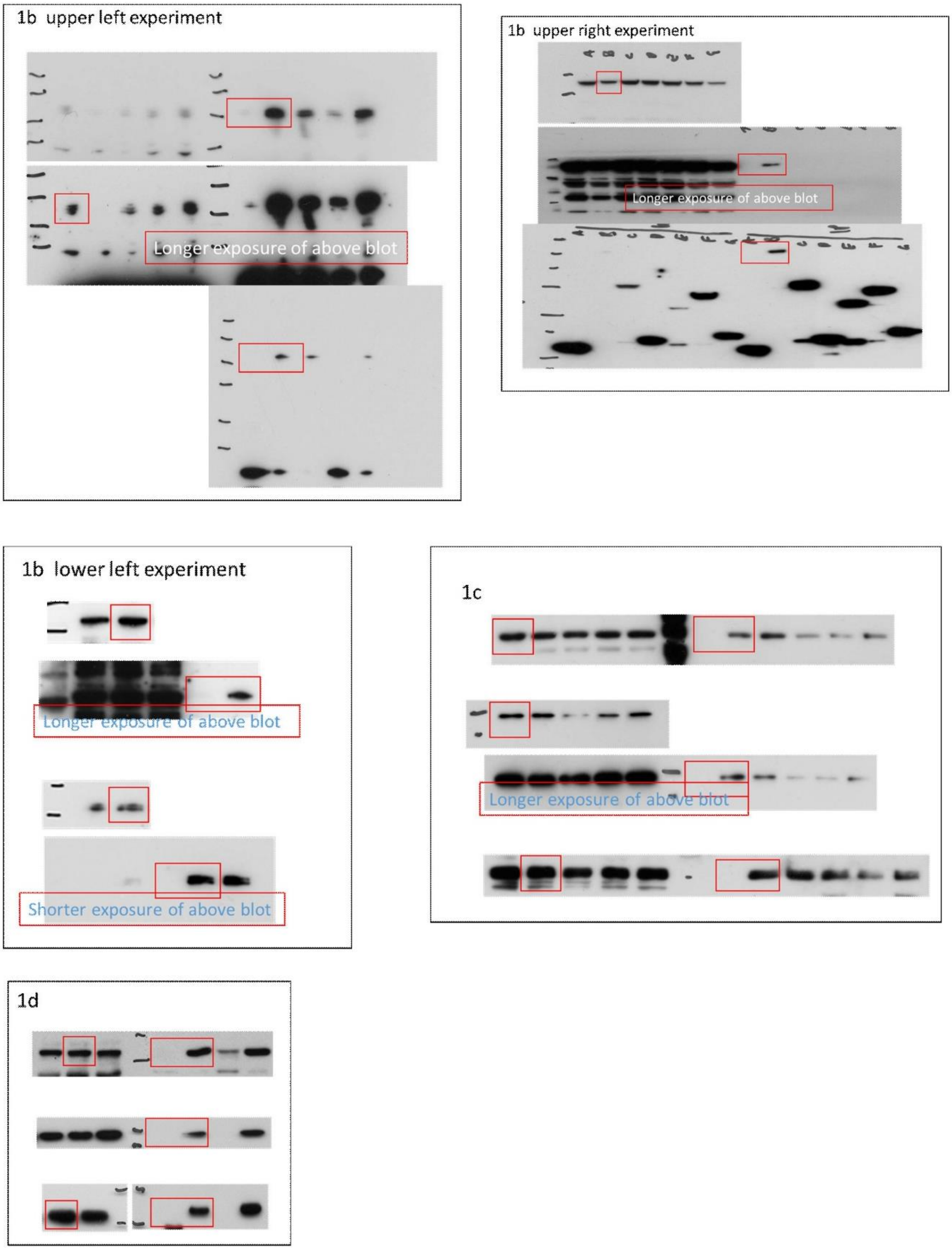

Supplementary figure 8. Uncropped blots for figure 1b-d

The uncropped blots displayed in figures 1b-d are presented. In some cases, the ‘total’ lane displays a different exposure of the same blot as the one presenting the immunoprecipitated (IP) samples, and this is indicated as appropriate. The cropped regions are denoted by red boxes.

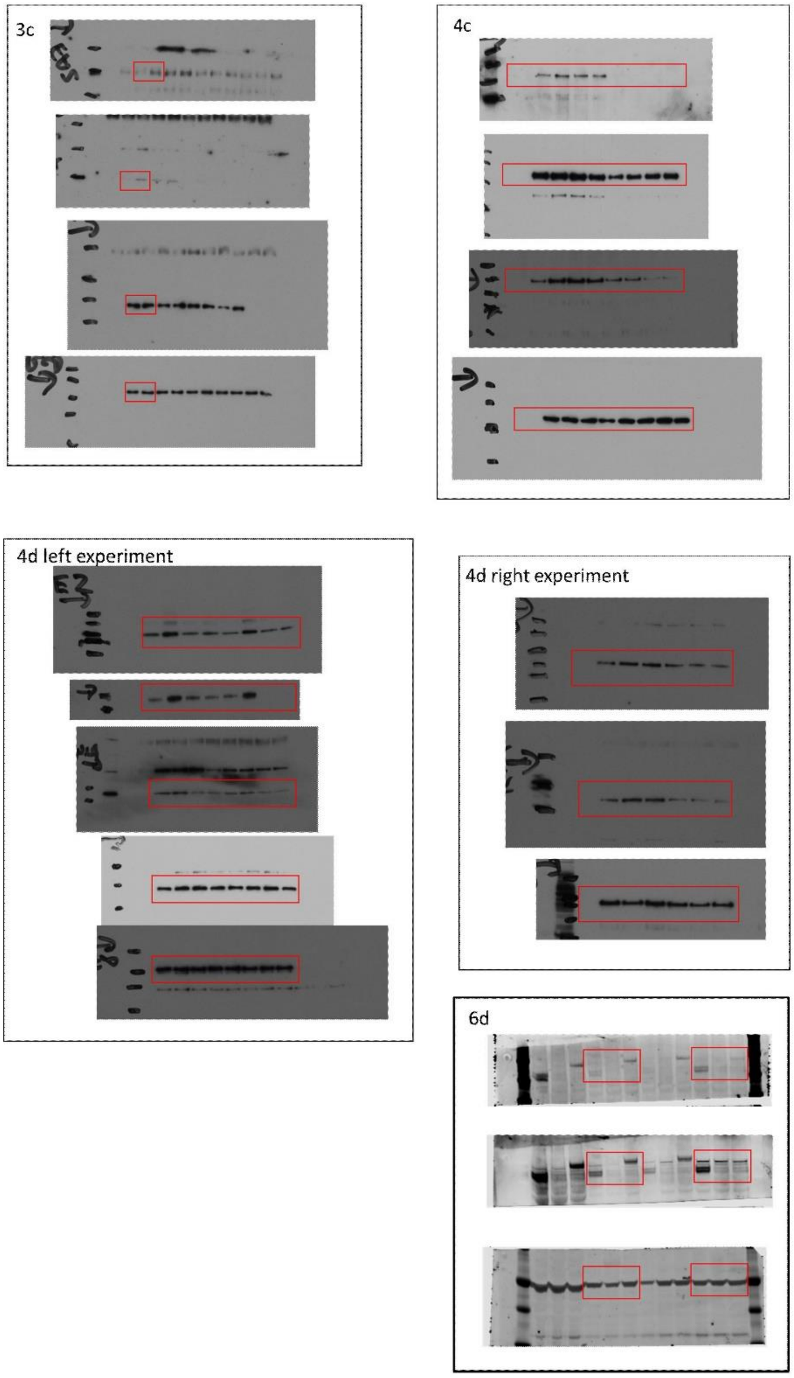

Supplementary figure 9. Uncropped blots for figures 3c, 4c-d & 6d

The uncropped blots displayed in figures 3, 4 & 6 are presented. The cropped regions are denoted by red boxes.
