# Supplementary material for: Phosphorylation of Rab-coupling protein by LMTK3 controls Rab14-dependent EphA2 trafficking to promote cell:cell repulsion
Source: Nat Commun. 2017 Mar 15;8:14646. doi: 10.1038/ncomms14646 (PMC5355957; doi:10.1038/ncomms14646)
Supplement: Peer Review File [file ncomms14646-s8.pdf]

Editorial Note: this manuscript has been previously reviewed at another journal that is not operating a transparent peer review scheme. This document only contains reviewer comments and rebuttal letters for versions considered at Nature Communications.
